# Astrocyte specification in the mouse septum is shaped by both developmental origin and local signals

**DOI:** 10.1038/s41593-025-02007-z

**Published:** 2025-07-28

**Authors:** Yajun Xie, Christopher M. Reid, Miguel Turrero Garcίa, Fiona Dale-Huang, Alejandro A. Granados, Yi Lu, Jiwen Li, Sarah M. Hanson, Walter R. Mancia Leon, Jonathan Liu, Manal Adam, Olivia Mosto, Angela O. Pisco, Ye Zhang, Arturo Alvarez-Buylla, Corey C. Harwell

**Affiliations:** 1 Department of Neurology, University of California, San Francisco, San Francisco, CA USA; 2 Eli and Edythe Broad Center of Regeneration Medicine and Stem Cell Research, San Francisco, CA USA; 3 Department of Neurobiology, Harvard Medical School, Boston, MA USA; 4 Ph.D. Program in Neuroscience, Harvard University, Boston, MA USA; 5 Department of Neurological Surgery, University of California, San Francisco, San Francisco, CA USA; 6 Chan Zuckerberg Biohub San Francisco, San Francisco, CA USA; 7 Department of Psychiatry and Biobehavioral Sciences, David Geffen School of Medicine, University of California, Los Angeles, Los Angeles, CA USA

**Keywords:** Glial development, Differentiation

## Abstract

Astrocyte specification during development is influenced by both intrinsic and extrinsic factors, but the precise contribution of each remains poorly understood. Here we show that mouse septal astrocytes derived from *Nkx2.1*- and *Zic4*-expressing progenitor zones are primarily allocated into the medial septal and lateral septal nuclei, respectively. Astrocytes in these areas exhibit distinctive molecular and morphological features. Using single-nucleus RNA sequencing, we traced the developmental trajectories of cells in the septum and found that neurons and astrocytes undergo region-specific and developmental-stage-specific local cell–cell interactions. Expression of the morphogens sonic hedgehog and fibroblast growth factors by medial septal and lateral septal neurons, respectively, promote the specification of astrocytes in each region. Finally, heterotopic cell transplantation studies showed that septal astrocyte specification depends on the local microenvironment, regardless of developmental origin. Our data highlight the importance of the local environment in determining astrocyte functional specialization.

## Abstract (web summary)

The relative contribution of intrinsic and extrinsic factors to astrocyte heterogeneity is unknown. Using heterotropic transplantation of septal astrocytes with distinct lineage origins, we show that local factors specify their ultimate identities.

## Main

The septal area, situated in the ventral forebrain, governs a diverse array of behaviors related to memory, movement, mood and motivational processes^1–4^. There has been substantial recent progress in delineating the roles of genetically defined septal neuron types^5–12^. However, there remains a gap in understanding the extent and functional relevance of glial cell diversity in the septum. It is now appreciated that glial cells notably contribute to neural circuit function and behavior outputs^13–16^. Therefore, it is important to understand the heterogeneity of glial cell types within the septal area, to complement our knowledge of the functional diversity of septal neurons.

Astrocytes are an abundant glial cell type extensively distributed throughout the central nervous system (CNS). There is a growing recognition of the morphological and molecular diversity exhibited by astrocytes in different CNS regions^17–21^. However, the developmental mechanisms underlying the diversification and functional specialization of astrocytes to support a wide range of neural circuits are not understood. It is widely recognized that astrocyte specification is shaped by the interplay of intrinsic factors related to developmental origins or progenitor lineages and extrinsic factors derived from the local cellular environment^22–24^. However, the precise roles and contributions of each of these factors have yet to be determined.

Here, by combining genetic fate mapping with single-nucleus RNA sequencing (snRNA-seq), we identified two distinct astrocyte populations residing in the medial septum (MS) and lateral septum (LS). Beyond their anatomical allocation, these two astrocyte populations are also distinguished by their developmental origins and their molecular and morphological properties. Many of the genes that distinguish MS and LS astrocytes are downstream transcriptional targets of sonic hedgehog (SHH) and fibroblast growth factor (FGF) signaling, two neuron-derived secreted factors expressed in the MS and LS, respectively. To determine the precise role of local environmental cues in specifying the distinctive features of MS and LS astrocytes, we performed heterotopic transplantations of cells derived from either lineage and found that transplanted astrocytes adopted morphological and molecular features of astrocytes in the host region, regardless of their developmental origin. Together, our findings indicate that neuron-derived factors have a profound impact on shaping the specialized properties of astrocytes within their local environment and exemplify the remarkable plasticity of astrocytes to adapt to their surroundings.

## Results

### Allocation of *Nkx2.1*- and *Zic*-derived septal astrocytes

**Fig. 1:**
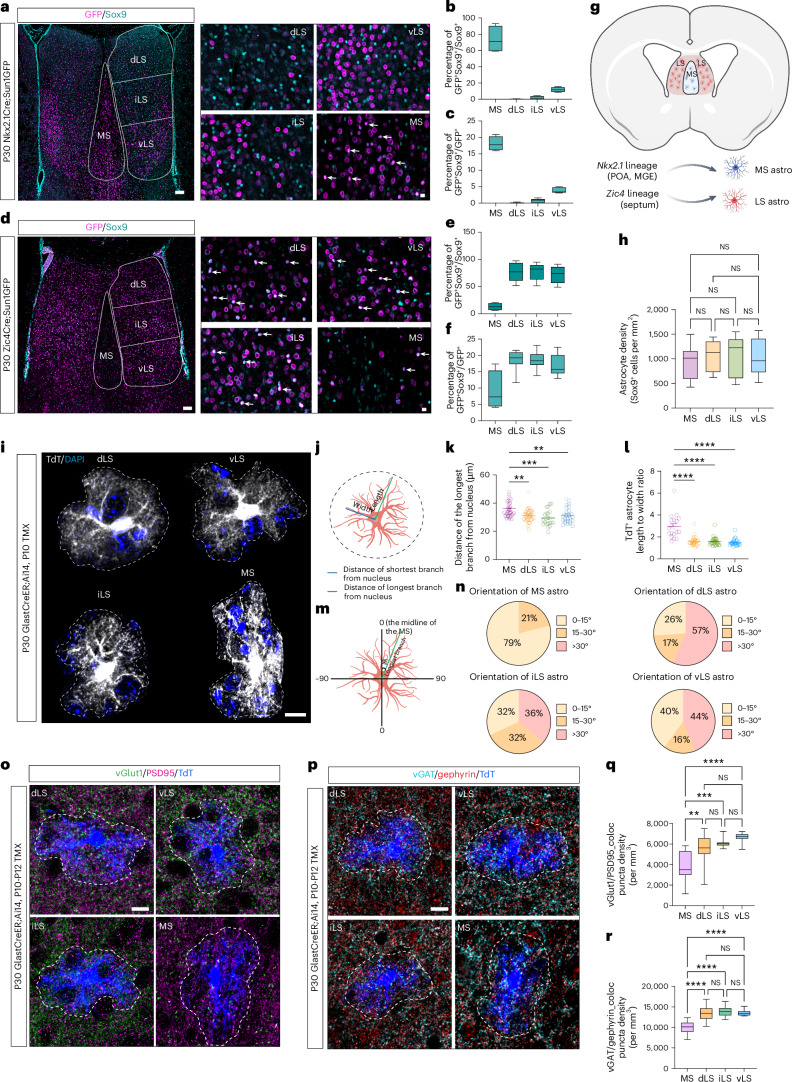
*Nkx2.1*- and *Zic4*-derived astrocytes occupy MS and LS, respectively. **a**, Immunostaining for GFP and Sox9 in P30 Nkx2.1Cre;Sun1GFP mice. The white arrows indicate cells with overlapping signals. Scale bars, 100 µm (left), 10 µm (right). **b**, Proportion of *Nkx2.1*-derived astrocytes within the total astrocyte population (*n* = 4–5 mice). **c**, Proportion of *Nkx2.1*-derived astrocytes within the total *Nkx2.1*-derived cells (*n* = 4–5 mice). **d**, Immunostaining for GFP and Sox9 in P30 Zic4Cre;Sun1GFP mice. The white arrows indicate cells with overlapping signals. Scale bars, 100 µm (left), 10 µm (right). **e**, Proportion of *Zic4*-derived astrocytes within the total astrocyte population (*n* = 3–5 mice). **f**, Proportion of *Zic4*-derived astrocytes within the total *Zic4*-derived cells (*n* = 3–5 mice). **g**, Schematic of astrocyte (astro) lineages in the septum. **h**, Astrocyte density in the septum (one-way analysis of variance (ANOVA) and Tukey’s multiple-comparison test, *n* = 9–11 mice). **i**, High magnification of representative tdT^+^ astrocytes with DAPI staining in the septum. Scale bar, 10 µm. **j**, Schematic of the methods for analyzing astrocyte length to width ratio. **k**, Analysis of the distance of the longest branch from the nucleus in the septum (one-way ANOVA and Tukey’s multiple-comparison test; *n* = 4 mice, total 20–29 astrocytes in each region). **l**, Analysis of the length to width ratio of tdT^+^ astrocytes (one-way ANOVA and Tukey’s multiple-comparison test; *n* = 4 mice, 19–25 astrocytes in each region). **m**, Schematic of the methods for analyzing astrocyte orientation. **n**, Pie charts of the percentage of astrocytes displaying angles in the septum (*n* = 4 mice, 19–25 astrocytes in each region). **o**, Immunostaining for vGlut1/PSD95 in P21 GlastCreER;Ai14 septum. Astrocyte territories are indicated with white dashed lines. Scale bar, 10 µm. **p**, Immunostaining for vGAT/gephyrin and tdTomato in P21 GlastCreER;Ai14 septum. Astrocyte territories are indicated with white dashed lines. Scale bar, 10 µm. **q**,**r**, Quantification of excitatory synapse (**q**) and inhibitory synapse (**r**) density in tdT^+^ astrocyte domains per mm^3^ (one-way ANOVA, Tukey’s multiple-comparison test; *n* = 3 mice, 8–12 astrocytes in each region). The box plots show the median (center), the box the 25th and 75th percentiles and the whiskers the minimum to maximum. The graphs show the mean ± s.e.m. **P < 0.01, ***P < 0.001, ****P < 0.0001. When *P* > 0.05, it is nonsignificant (NS). Icons in **g** created with BioRender.com. Source data

**Extended Data Fig. 1:**
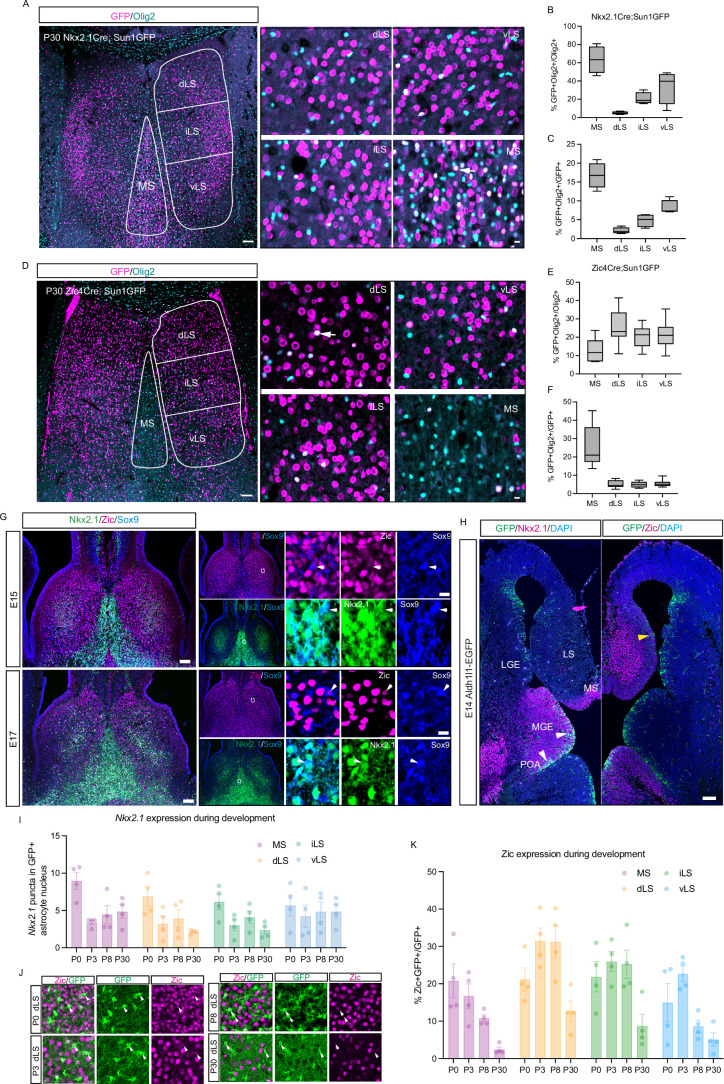
Nkx2.1 and Zic4 lineages-derived astrocytes occupy MS and LS respectively. **a**) Immunostaining for Olig2 (cyan) and GFP (magenta) in P30 Nkx2.1Cre; Sun1GFP septum. Subdivisions of the septum are indicated with white lines. White arrows indicate *Nkx2.1*-derived oligodendrocytes. Scale bars: left: 100 µm, right: 10 µm. **b**) Proportion of *Nkx2.1*-derived oligodendrocytes in total oligodendrocytes. N = 4 mice. **c)** Proportion of *Nkx2.1*-derived oligodendrocytes in total Nkx2.1-derived cells. N = 4 mice. **d**) Immunostaining for GFP (magenta) and Olig2 (cyan) in P30 Zic4Cre; Sun1GFP mice. Subdivisions of the septum are indicated with white lines. White arrows indicate cells with overlapping signals. Scale bars: left: 100 µm, right: 10 µm. **e**) Proportion of Zic4-derived oligodendrocytes in total oligodendrocytes. N = 5 mice. **f)** Proportion of Zic4-derived oligodendrocytes in total Zic4-derived cells. N = 5 mice. **g**) Immunostaining for Nkx2.1 (green), Zic (magenta), and Sox9 (blue) in coronal sections of E15 and E17 CD1 mice. White boxes indicate the selected regions for high magnification images (right panels. White arrows indicate the Zic + /Sox9+ and Nkx2.1 + /Sox9+ cells. Scale bars: left: 100 µm, right: 10 µm. **h**) Immunostaining for GFP (green), Nkx2.1 (magenta), Zic (magenta), and DAPI (blue) in horizontal sections of E14 Aldh1l1-EGFP mice. Lateral ganglionic eminence (LGE); medial ganglionic eminence (MGE); preoptic area (POA). White arrows indicate the Nkx2.1+ /GFP+ astrocyte progenitors and a yellow arrow indicates Zic+ /GFP+ astrocyte progenitors. Scale bar: 100 µm. **i**) RNAscope for *Nkx2.1* in the septum of Aldh1l1-EGFP mice, and quantification of *Nkx2.1* puncta in GFP+ astrocytes during postnatal developments. N = 4 mice for each age. **j**) Representative images showing immunostaining for Zic in postnatal dLS astrocytes. GFP positive cells represent astrocytes in the Aldh1l1-EGFP mouse line. White arrows indicate astrocytes with low levels of Zic. Scale bar: 20 µm. **k**) Quantification of the percentage of Zic+GFP + /GFP+ cells in the septum of Aldh1l1-EGFP mice, and. N = 4 mice for each age. Box plots in B, C, E and F show the median (center), 25th and 75th percentiles (box), and min to max (whiskers). Bar graphs in I, K show the mean ± s.e.m. Source data

Nkx2.1 and Zic transcription factors are highly expressed in distinct progenitor domains during embryonic stages^5,7,25,26^. To determine the specific contribution of Nkx2.1 progenitors to septal astrocyte diversity, we used the Nkx2.1-Cre mouse line crossed with the Sun1GFP reporter line to label cells with a history of *Nkx2.1* expression. Immunostaining the septum of postnatal day (P) 30 Nkx2.1-Cre;Sun1GFP mice with the astrocyte marker Sox9 revealed that most Sox9^+^ astrocytes (74%) in the MS have a history of *Nkx2.1* expression^5,12^. In contrast, there are fewer *Nkx2.1*-derived astrocytes in subdivisions of the LS (dorsal (d)LS: 0.1%; intermediate (i)LS: 2%; and ventral (v)LS: 12%; Fig. 1a,b), mirroring oligodendrocyte lineage patterns (Extended Data Fig. 1a,b). Sun1GFP-positive astrocytes in the MS make up 18% of all *Nkx2.1*-derived cells (Fig. 1c), in addition to other neuronal and glial cell types within the MS^5,7^ (Extended Data Fig. 1c). To determine the contribution of Zic-expressing progenitors to astrocytes in the septum, we generated a Zic4Cre;Sun1GFP reporter mouse line. Our staining indicated that LS, but not MS, astrocytes are mainly derived from *Zic4*-expressing progenitors (dLS: 77%; iLS: 78%; vLS: 71%; and MS: 13%) and make up <20% of all Zic4 lineage cells (Fig. 1d–f and Extended Data Fig. 1d–f)^5–7,27^. We stained for Sox9 combined with lineage markers in both wild-type (CD1) mice and the astrocyte reporter line Aldh1l1Cre-EGFP, and observed that astrogenesis is already present in MS and LS, respectively, as early as embryonic day (E)14 (Extended Data Fig. 1g,h). *Nkx2.1*-expressing astrocyte progenitors are mainly located in the medial ganglionic eminence (MGE) and preoptic area (POA), whereas the *Zic*-expressing astrocyte progenitors are enriched in the embryonic septum^28–30^. Taken together, our data suggest that MS and LS astrocytes are mostly derived from distinct progenitor domains (Fig. 1g). We found that *Nkx2.1* expression in astrocytes becomes almost undetectable after birth (Extended Data Fig. 1i). Similarly, Zic protein levels in astrocytes remain relatively low during postnatal development (Extended Data Fig. 1j,k). This suggests that these transcription factors likely influence earlier events in the production and allocation of astrocytes, rather than their differentiation after birth^28,31,32^.

**Extended Data Fig. 2:**
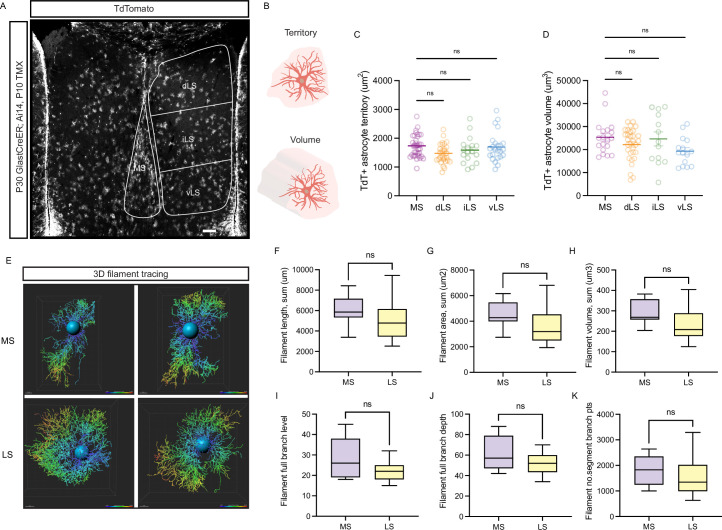
Astrocyte morphological properties in the septum. **a)** Immunostaining for tdTomato (tdT + ) (grey) in the septum of P21 GlastCreER; Ai14 mice injected intraperitoneally with tamoxifen at P10. Scale bar: 100 µm. **b**) Schematic of the methods for analyzing astrocyte territory and volume. **c**) Analysis of the territories of tdT+ astrocytes (µm^2^) (One-way ANOVA, Tukey’s multiple comparisons test, N = 4 mice, with a total of 17-36 astrocytes analyzed per region). **d**) Analysis of the volume of TdT+ astrocytes in different septal regions (One-way ANOVA, Tukey’s multiple comparisons test, N = 4 mice, with a total of 13-31 astrocytes analyzed per region). **e**) Representative 3D filament tracings of MS and LS astrocytes using Imaris. **f**-**k**) Analysis of septal astrocyte filament length (sum, in µm) (**f**), filament area (sum, in µm^2^) (**g**), filament volume (sum, in µm^3^) (**h**), filament full branch level (**i**), filament full branch depth (**j**) and filament number of segment branch points (**k**) (two-tailed, unpaired t-test with Welch’s correction, N = 4 mice, with a total of 11-12 astrocytes per region for filament tracing and analysis). Graphs in C, D show the mean ± s.e.m. Box plots in F-K show the median (center), 25th and 75th percentiles (box), and min to max (whiskers). *p < 0.05, **p < 0.01, ***p < 0.001, ****p < 0.0001. When the p-value was greater than 0.05, it was stated as non-significant (ns). Source data

To investigate the cellular properties of MS and LS astrocytes, we used a GlastCreER;Ai14 reporter mouse line induced with tamoxifen at P10 (Extended Data Fig. 2a). Astrocytes in the MS can be distinguished from those in the LS by their longer branches (Fig. 1i–l and Extended Data Fig. 2e) and a more polarized distribution of branch angles along the axis of the midline compared with LS astrocytes (Fig. 1m,n), aligning with the dense axon tracts traversing the MS^33^. However, the territory size, volume, density and branch complexity of septal astrocytes did not significantly differ (Fig. 1h and Extended Data Fig. 2b–k). We examined septal astrocyte morphology and tiling over development and observed that astrocytes are distributed evenly across the cytoarchitectural boundaries segregating the MS and LS (Supplementary Fig. 1a,b). This suggests that astrocytes uniformly tile the septum regardless of the anatomical regions that they occupy. Further staining showed that MS astrocytes closely interact with local parvalbumin-positive neurons, whereas LS astrocytes wrap adjacent calbindin-positive neurons (Supplementary Fig. 1c,d). Neurons in the septum receive inputs from a diversity of excitatory and inhibitory projection neurons from other brain regions. Hippocampal neurons provide a major source of glutamatergic inputs into the LS, whereas MS neurons receive mostly γ-aminobutyrate-ergic (GABA-ergic) inputs from LS and hippocampal neurons^34–41^. To investigate synapse interactions within astrocyte territories, we stained for vGlut1/PSD95 and vGAT/gephyrin, which identify glutamatergic and GABA-ergic synapses, respectively, in P30 GlastCreER;Ai14 mice (Fig. 1o,p). There were significantly fewer glutamatergic and GABA-ergic colocalized puncta within MS astrocyte territories compared with the LS (Fig. 1q,r). These data collectively suggest that MS and LS astrocytes are derived from distinct developmental lineages and exhibit unique morphologies and synaptic interactions suited to the specific neuronal circuits that they support.

### Molecular heterogeneity of septal astrocyte lineages

**Fig. 2:**
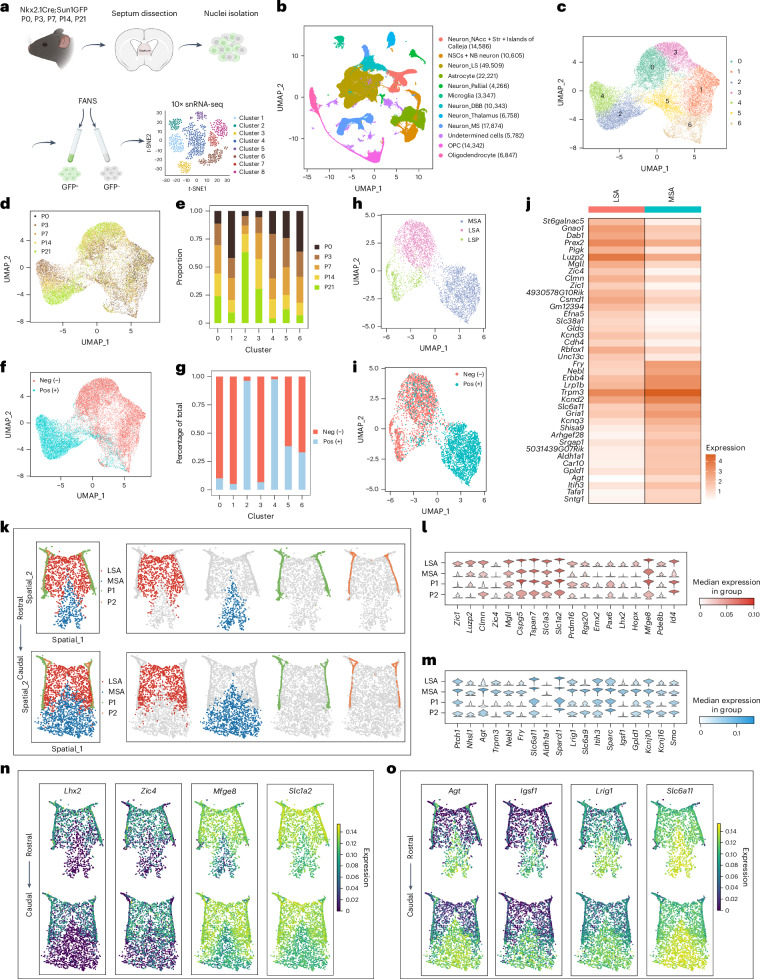
Septal astrocytes display distinct lineage-specific molecular signatures. **a**, Experimental design for snRNA-seq on sorted cells from Nkx2.1Cre;Sun1GFP septum at different developmental stages. FANS, fluorescence-activated nuclei sorting. **b**, Uniform Manifold Projection and Approximation (UMAP) plot of cell types from all samples (*n* = 166,418 cells). **c**, UMAP plot of all septal astrocyte clusters. **d**, UMAP plot of septal astrocytes annotated by age. **e**, Proportion of cells at each age across clusters. **f**, UMAP plot of all GFP^+^ and GFP^−^ astrocytes. **g**, Proportion of GFP^+^ and GFP^−^ astrocytes in each cluster. **h**, UMAP plot of P21 septal astrocytes at a 0.3 resolution level. **i**, UMAP plot of P21 GFP^+^ and GFP^−^ astrocytes. **j**, Heatmap showing the top 20 DEGs in LSAs and MSAs. **k**, MERSCOPE analysis of P35 septal astrocyte clusters along the rostrocaudal axis. **l**,**m**, MERSCOPE analysis showing gene enrichment in LSAs (**l**) and MSAs (**m**). **n**,**o**, MERSCOPE analysis showing representative genes enriched in LSAs (**n**) and MSAs (**o**) along the rostrocaudal axis. Icons in **a** created with BioRender.com. LSP1, lateral septal astrocyte progenitor cluster 1; LSP2, lateral septal astrocyte progenitor cluster 2; neg, negative; pos, positive; *t*-SNE, *t*-distributed stochastic neighbor embedding.

**Extended Data Fig. 3:**
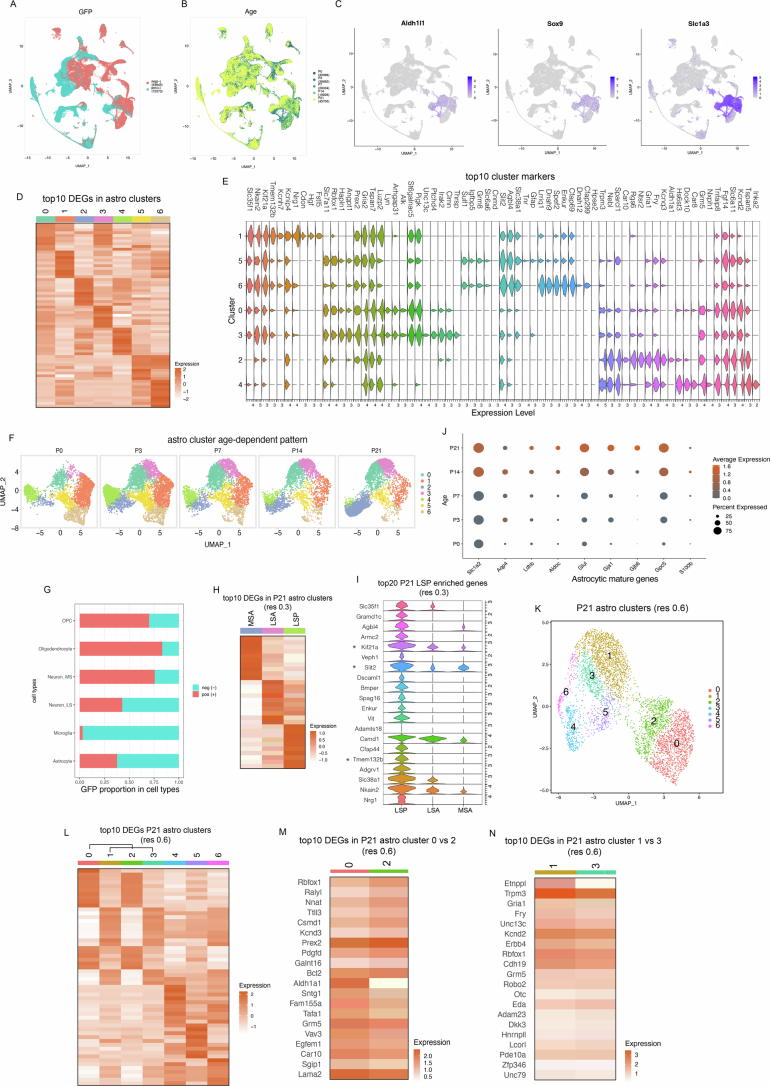
Septal astrocytes display lineage-specific molecular profiles. **a**) UMAP plot of *Nkx2.1*-derived (GFP+) and non-*Nkx2.1*-derived (GFP-) cells. **b**) UMAP analysis of total cells at different ages. **c**) Feature plots showing astrocyte populations labeled with general astrocytic markers: *Aldh1l1*, *Sox9* and *Slc1a3*. **d)** Heatmap showing the top 10 DEGs in seven subgroups of astrocytes. **e**) Top 10 DEGs enriched in individual astrocyte clusters. **f**) Dynamic changes in subsets of astrocyte populations over age. **g**) Proportion of GFP+ and GFP- cells in each cell type. **h**) Heatmap showing the top 10 DEGs in P21 astrocyte clusters at a 0.3 resolution level. **i**) A violin plot showing the top 20 genes highly enriched in the LSP cluster. * indicates genes highlighted in the text. **j**) Expression of general mature astrocytic genes in septal astrocytes across developmental stages. **k**) UMAP plot showing 7 astrocyte subclusters at P21 at a 0.6 resolution level. **l**) Heatmap showing the top 10 DEGs in seven subgroups of P21 astrocytes at a 0.6 resolution level. **m**) Heatmap showing the top 10 DEGs in P21 astrocyte cluster 0 versus cluster 2 (corresponding to Extended Data Fig. 3K) at a 0.6 resolution level. **n**) Heatmap showing the top 10 DEGs in P21 astrocyte cluster 1 versus cluster 3 (corresponding to Extended Data Fig. 3K) at a 0.6 resolution level.

The distinctive developmental origins and cellular features of MS and LS astrocytes suggest that they may possess additional molecular specializations. To investigate this further, we collected the septa of Nkx2.1Cre;Sun1GFP mice at five developmental stages between P0 and P21. To distinguish cells with a history of Nkx2.1 expression from other lineages, we isolated GFP^+^ and GFP^−^ nuclei at each time point and performed snRNA-seq (Fig. 2a). We collected a total of 166,418 nuclei (Fig. 2b) from both Nkx2.1 (GFP^+^) and non-Nkx2.1 (GFP^−^) lineages (Extended Data Fig. 3a) across all time points (Extended Data Fig. 3b). A total of 22,221 astrocytic nuclei (Fig. 2c) were classified into 7 distinct subpopulations based on their transcriptional profiles (Extended Data Fig. 3c–f). Clusters 1, 5 and 6 primarily represented early lateral ventricular progenitors that decrease in abundance over time^42–44^ (Extended Data Fig. 3e,f). Based on both GFP expression (Fig. 2f,g) and the dynamic changes observed in these clusters over time (Fig. 2d,e and Extended Data Fig. 3f), clusters 0 and 3 were identified as LS astrocytes, whereas clusters 4 and 2 corresponded to early and late-stage MS astrocytes, respectively. The clusters corresponding to astrocytes and neurons exhibited a clear segregation based on developmental origin (Extended Data Fig. 3a,g). To investigate the molecular differences between late-stage MS and late-stage LS astrocytes, we subclustered P21 astrocytes and identified three distinct clusters, including late-stage MS (GFP^+^) astrocytes (MSAs), LS (GFP^−^) astrocytes (LSAs) and one lateral septal or ventricular progenitor cluster (LSP)^42–44^ (Fig. 2h,i and Extended Data Fig. 3h,i). The top 20 differentially expressed genes (DEGs) in MSA and LSA populations revealed robust lineage-specific segregation in their molecular profiles, including the lineage-specific enrichment of *Zic* family members in GFP^−^ astrocytes (Fig. 2j). P21 astrocytes show enrichment of mature marker genes, suggesting their maturity by this time point^45,46^ (Extended Data Fig. 3j). Further clustering did not reveal substantial differences in the top DEGs, suggesting that astrocyte molecular diversity is primarily defined by lineage (Extended Data Fig. 3k–n). Molecular similarities among astrocyte types are likely closely linked to their regional proximity to each other in the brain^47^; thus, to further elucidate these relationships, we integrated our P21 snRNA-seq astrocytes with scRNA-seq data from cortical, hippocampal and striatal astrocytes^47^, which are neighbors to septal astrocytes. Our data showed that both MSA and LSA clusters are distinct from astrocytes in other regions based on their molecular signatures (Supplementary Fig. 2a–c).

Further analysis showed that LSAs expressed genes highly enriched in cortical and hippocampal astrocytes, whereas MSAs were enriched in genes specific to midbrain or hindbrain astrocytes^47^ (Supplementary Fig. 2d,e). This suggests that septal astrocytes share some common transcriptional features with astrocytes from other brain regions. To visualize the spatial distribution of astrocyte populations in the septum, we performed single-cell spatial transcriptomics (MERFISH) on the septum at P35, with a list of 500 genes enriched in defined astrocyte and neuron clusters. We identified 25 clusters, including both astrocyte and neuron subpopulations displaying differential spatial distributions in the septum (Supplementary Fig. 3a,b), based on their general markers (Supplementary Fig. 3c,e)^10^. We selected astrocyte populations for further analysis and identified four astrocyte clusters, including LS, MS and two ventricular zone progenitor subgroups based on the expression of cluster-enriched genes (Fig. 2k–m and Supplementary Fig. 3d), consistent with our snRNA-seq data (Fig. 2j and Extended Data Fig. 3h). Some genes exhibited expression gradients from high to low between LSAs and MSAs (for example, *Mfge8* and *Slc1a2*), or vice versa (for example, *Lrig1* and *Slc6a11*), whereas others were exclusively expressed by LSAs (for example, *Lhx2* and *Zic4*) or MSAs (for example, *Agt* and *Igsf1*) (Fig. 2n,o). Together, these data illustrate that MSAs and LSAs exhibit distinct molecular profiles that correspond to their developmental origins.

### Developmental trajectories of MSAs and LSAs

**Fig. 3:**
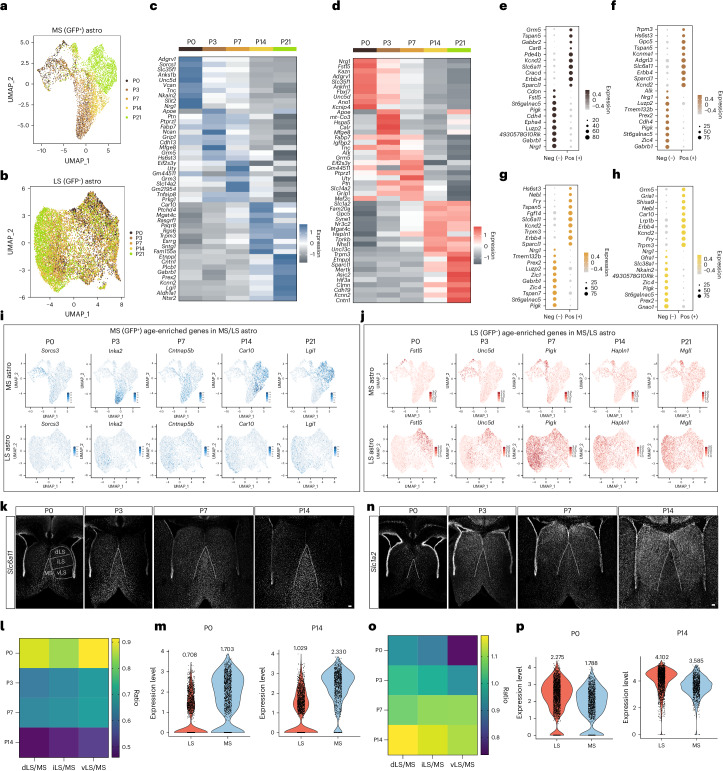
MSAs and LSAs display unique age-dependent molecular profiles. **a**,**b**, UMAP plot showing age-dependent MSA (**a**) and LSA (**b**) clusters. **c**,**d**, Heatmap showing the top-ten DEGs in GFP^+^ (**c**) and GFP^−^ (**d**) astrocytes across ages. **e**–**h**, Heatmaps showing the top-ten DEGs enriched in GFP^+^ versus GFP^−^ at P0 (**e**), P3 (**f**), P7 (**g**) and P14 (**h**). **i**, Representative genes enriched in GFP^+^ astrocytes during development. **j**, Representative genes enriched in GFP^−^ astrocytes during development. **k**, *Slc6a11* messenger RNA (mRNA) in P0–P14 septum of WT (CD1) mice detected using in situ hybridization. Subdivisions of the septum are indicated with white lines. Scale bar, 100 µm. **l**, Heatmap showing the relative density of puncta of *Slc6a11* mRNA in each corresponding LS subnucleus, normalized by the density of puncta in the MS, at different ages (*n* = 3–5 mice for each age). **m**, Violin plots showing *Slc6a11* expression (snRNA-seq) in both MSAs and LSAs at P0 and P14. The average expression levels are indicated. **n**, *Slc1a2* mRNA evaluated in P0–P14 septum of WT (CD1) mice using in situ hybridization. Scale bar, 100 µm. **o**, Heatmap showing the relative density of puncta of *Slc1a2* mRNA in each corresponding LS subnucleus, normalized by the density of puncta in the MS, at different ages (*n* = 3–5 mice for each age). **p**, Violin plots showing *Slc1a2* expression (snRNA-seq) in both MSAs and LSAs at P0 and P14. The average expression levels are indicated.

**Extended Data Fig. 4:**
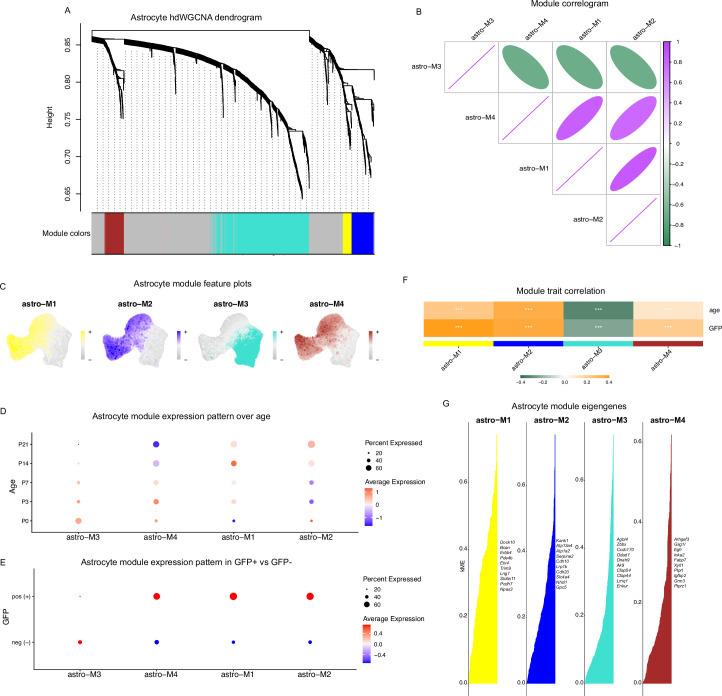
WGCNA analysis of astrocyte snRNA-seq dataset. **a**) Dendrogram analysis identifying four modules in the entire astrocyte population, including turquoise, blue, red and yellow modules. **b**) Correlation analysis of individual modules with each other. **c**) Feature plots displaying each astrocyte module. **d**) Module expression patterns across developmental stages. **e**) Module expression patterns in GFP+ and GFP- lineages. **f**) Statistics analysis of age-dependent and lineage-related module trait correlations. **g**) Module eigengenes analysis for each module.

**Extended Data Fig. 5:**
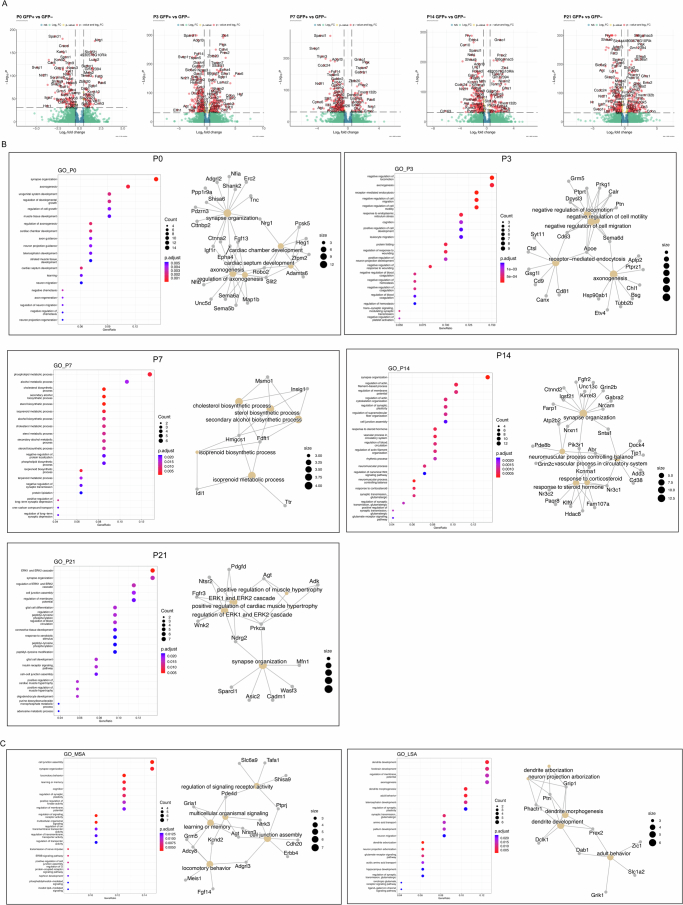
Age-dependent molecular profiles and function in septal astrocytes. **a**) Volcano plots showing the genes enriched in GFP+ versus GFP- developing astrocytes (Wilcoxon Rank-Sum Test for assessing the ranks of gene expression values differ significantly between conditions and Benjamini-Hochberg adjustment for multiple testing). **b**) Gene ontology analysis (top 20 gene terms) of DEGs in septal astrocytes at different ages. **c**) Gene ontology analysis (top 20 gene terms) of DEGs in P21 MS and LS astrocytes. Gene ontology analysis is performed in R by using enrichGO() (v4.6.2). Hypergeometric test for GO term enrichment, and Benjamini-Hochberg adjustment for multiple testing.

Developing astrocytes proceed through a series of transitional states influenced by intrinsic and extrinsic factors^22,23^. Our analysis revealed clear and unique trajectories in both MSAs and LSAs during their development (Fig. 3a,b). To understand the transcriptional programs associated with the maturation of these regional astrocytes, we examined the top ten temporally enriched genes for both MSAs and LSAs (Fig. 3c,d) and compared differential gene expression of MSAs versus LSAs at P0, P3, P7 and P14 (Fig. 3e–h and Extended Data Fig. 5a). MSAs and LSAs exhibited lineage-associated developmental gene expression programs (Fig. 3i,j). To assess whether astrocyte clusters are primarily defined by lineage or age, we performed a weighted gene co-expression network analysis (WGCNA) on the entire astrocyte population. We identified four distinct modules based on gene enrichment patterns (Extended Data Fig. 4a–c). Modules M1 and M4 are more associated with lineage than age, whereas M3 is negatively correlated with both age and Nkx2.1 (GFP^+^) lineage and M2 shows a positive correlation with both (Extended Data Fig. 4d–f). Analysis of module eigengenes identified genes strongly associated with lineage in M1, including *Erbb4*, *Lrig1* and *Slc6a11* (Extended Data Fig. 4g). Collectively, these data suggest that the molecular identity of septal astrocytes is predominantly determined by lineage rather than age. To further assess the function of age-enriched genes, we performed gene ontology (GO) on septal astrocytes at each age. During early postnatal time points (P0–P3), GO terms related to synapse organization and axonogenesis were significantly represented, whereas, at P7, GO terms were predominantly associated with metabolic processes. At later postnatal time points (P14–P21), GO terms were correlated with cell junction and synapse assembly (Extended Data Fig. 5b). Differences in biological processes between MSAs and LSAs were evident, with the top genes enriched in P21 MSAs linked to cell junction assembly and organization, whereas gene terms in LSAs were associated with dendrite development and glutamatergic synaptic transmission, consistent with the major glutamatergic inputs from the hippocampus to the lateral septum^48,49^ (Extended Data Fig. 5c).

**Extended Data Fig. 6:**
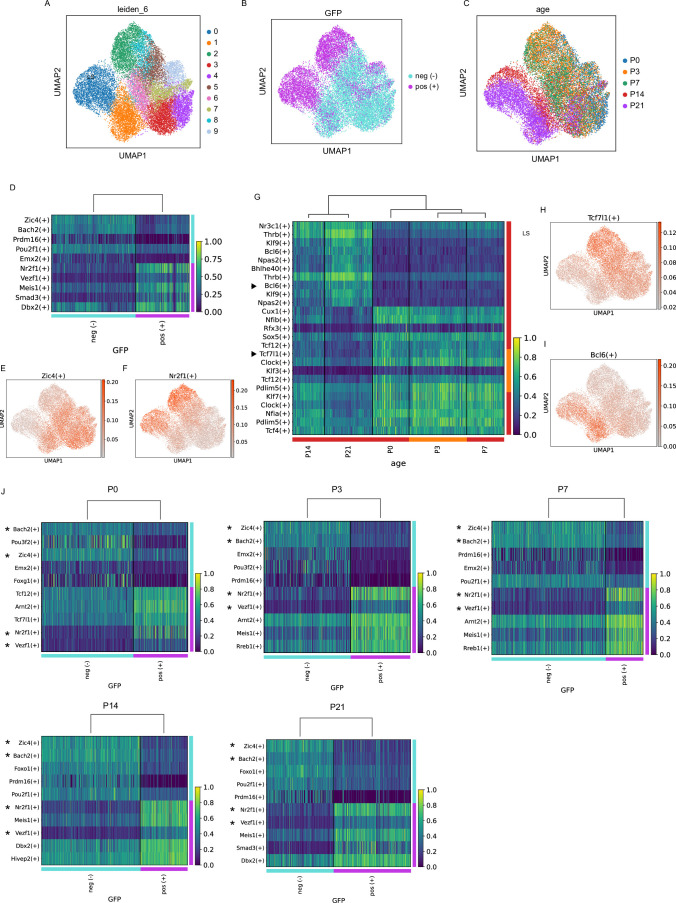
SCENIC analysis for regulon networks in septal astrocytes. **a**) UMAP plot defines 10 subclusters of astrocytes regarding distinct regulon networks. **b**) GFP+ and GFP- astrocytes are distinguished from each other based on regulon networks. **c**) Age-dependent regulon networks in septal astrocytes through development. **d**) Top 5 regulons enriched in MS and LS astrocytes. **e, f**) Feature plots showing Zic4 (**e**) and Nr2f1 (**f**) regulons highly enriched in LS and MS astrocytes respectively. **g**) Top enriched age-dependent regulons in septal astrocytes. Arrows indicate the highlighted genes in the text. **h, i**) Tcf7l1 (**h**) and Bcl6 (**i**) are enriched in septal astrocytes at early and late ages. **j**) Top 5 enriched regulons in GFP+ and GFP- astrocytes at each age. * indicates the conserved regulons at different ages.

Mapping gene families responsible for circuit-related functions^47^ to our P21 astrocyte clusters, we identified differential expression of K^+^ homeostasis and neurotransmitter transporters in both MSAs and LSAs (Supplementary Fig. 4), reflecting their interactions with different neural circuits^48–50^. Next, we used single-cell regulatory network inference and clustering (SCENIC) analysis^51^ to infer the transcription factor–target regulatory networks controlling lineage-specific and age-dependent astrocyte specification in the MS and LS. We identified ten regulatory network clusters associated with both lineage and age (Extended Data Fig. 6a–c). Regulons including *Zic4* were enriched in LSAs, whereas *Nr2f1* regulons were prominent in MSAs (Extended Data Fig. 6d–f). SCENIC analysis revealed several genes enriched in LSAs and MSAs that were inferred to be regulated by *Zic4* and *Nr2f1*, respectively (Supplementary Table 1). Certain regulons were temporally enriched such as *Tcf7l1* (P0–P7) and *Bcl6* regulons (P14–P21) (Extended Data Fig. 6g–i), whereas other regulons remained enriched throughout postnatal development (Extended Data Fig. 6j). These results suggest that septal astrocytes exhibit lineage-specific and age-dependent regulatory network enrichment. To further analyze the dynamic spatial and temporal expression patterns of region-specific genes in septal astrocytes, we studied the expression of the genes, respectively, encoding for GABA and glutamate transporters, *Slc6a11* and *Slc1a2*, by in situ hybridization in the septum at P0, P3, P7 and P14 (refs. ^52,53^). We observed that differential expression of *Slc6a11* and *Slc1a2* increased between MSAs and LSAs over time, with a comparable level in early stages that significantly diverged over development (Fig. 3k,l,n,o), consistent with our snRNA-seq analysis (Fig. 3m,p). This highlights the refinement of gene expression in septal astrocytes with age and suggests the potential influence of local environments on astrocyte molecular specification. Collectively, these data demonstrate that MSAs and LSAs undergo unique molecular specification during development, associated with their lineage origin and regional identity, ultimately leading to the expression of specialized circuit-related functional gene programs.

### Neuron-derived cues shape astrocyte features

**Fig. 4:**
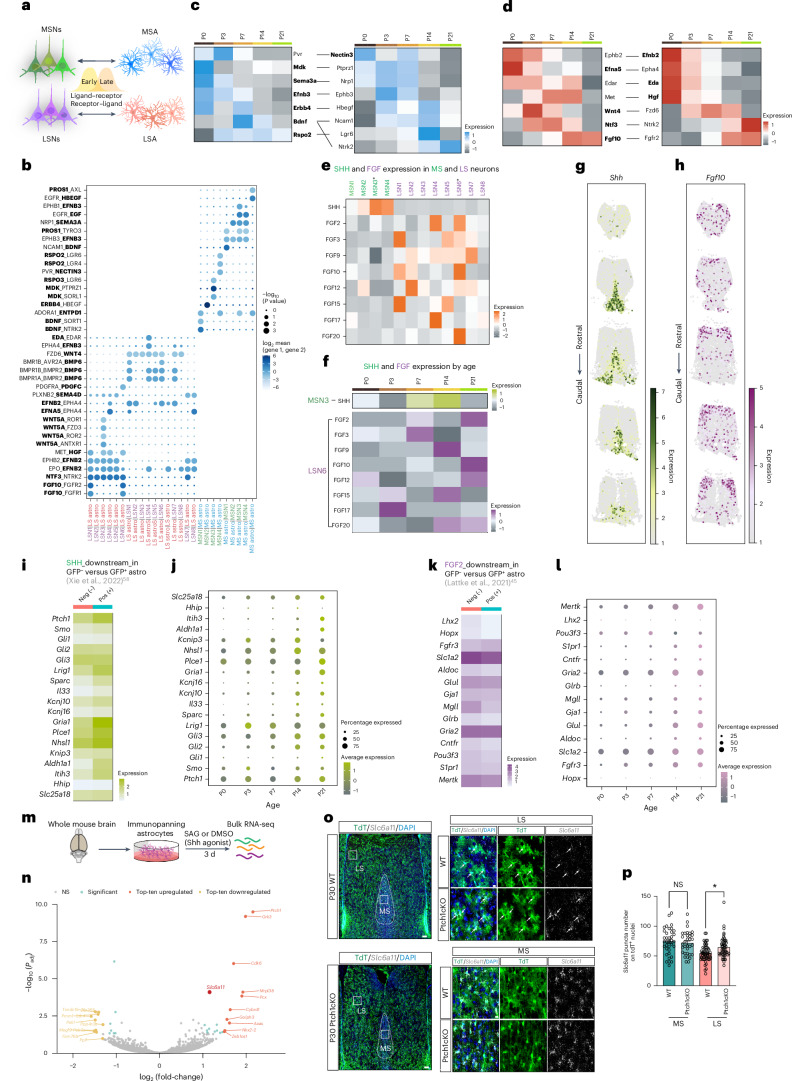
Region-specific and age-dependent interactions between septal astrocytes and neurons. **a**, Diagram of putative astrocyte–neuron interactions during development. **b**, CellPhoneDB analysis revealing exclusive ligand–receptor pairs between subclusters of LSNs (purple) with LSAs (red) and subclusters of MSNs (green) with MSAs (blue). Ligands are highlighted in bold (one-sided Wilcoxon’s rank-sum test to assess the statistical significance of each interaction score). **c**,**d**, Age-dependent ligand–receptor pairs in MS (**c**) and LS (**d**) neurons and astrocytes. Ligands are highlighted in bold. **e**, Heatmap showing that SHH and FGFs are highly enriched in MSN and LSN subclusters. Asterisks indicate the subclusters most enriched for SHH and FGF family ligands. **f**, Heatmap showing an age-dependent increase in the expression of SHH and FGFs in MSN3 and LSN6 subclusters, respectively. **g**,**h**, MERSCOPE showing SHH (**g**) and FGF10 (**h**) expression patterns in P35 septal neurons along the rostrocaudal axis. **i**,**k**, Heatmap showing selected SHH (**i**) or FGF2 (**k**) downstream genes enriched in P21 MSAs or LSAs. **j**, Dotplot showing increases in the expression of SHH downstream genes during development. **l**, Dotplot showing increases in the expression of FGF2 downstream genes during development. **m**, Schematic of the methods for bulk RNA-seq of immunopanned astrocytes treated with the SHH agonist SAG. **n**, Volcano plot illustrating differential gene expression between SAG and dimethyl sulfoxide (DMSO) treatments. For DESeq2 analysis, Wald’s test was used to assess the differences between conditions and the Benjamini–Hochberg method for multiple testing (*n* = 3–4 sample pools for each condition). **o**, In situ hybridization of *Slc6a11* combined with immunostaining for tdTomato in P21 tamoxifen-induced GlastCreER;Ai14 (WT) and GlastCreER;Ptch1fl/fl;Ai14 (Ptch1cKO) mice. Right: the white boxes indicate the selected regions for high-magnification images. The white arrows indicate the recombined astrocytes. Scale bars, 100 µm (left); 10 µm (right). **p**, Quantification of *Slc6a11* puncta number on tdT^+^ astrocyte nuclei (one-way ANOVA and Tukey’s multiple-comparison test; *n* = 4 mice, 33–59 astrocytes in each condition). Data are presented as the mean ± s.e.m. *P < 0.05. When *P* > 0.05, it is NS. Source data

**Extended Data Fig. 7:**
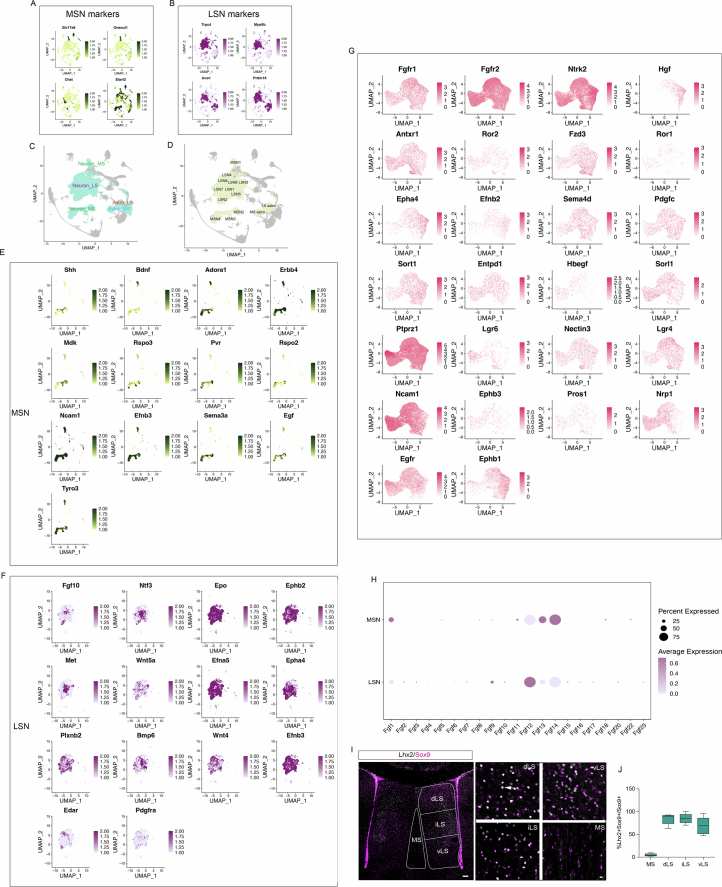
Ligand-receptor expression patterns in the septum. **a, b**) Representative genes that define MS (**a**) and LS (**b**) neurons. MSN: medial septal neuron; LSN: lateral septal neuron. **c**, **d**) Highlighted neuron (**c**) and astrocyte (**d**) subclusters that were processed by CellphoneDB analysis. **e-g**) Expression patterns of ligands and receptors in MSN (**e**), LSN (**f**), and septal astrocytes (**g**). **h**) Expression patterns of Fgf family members in septal neurons. **i**) Immunostaining for Lhx2 (grey) and Sox9 (magenta) in P30 wild-type septum. Subdivisions of the septum are indicated with white lines. The white arrow indicates a cell with overlapping signals. Scale bars: left, 100 µm; right, 10 µm. **j**) Proportion of Lhx2+ astrocytes in total astrocytes across different regions. N = 5 mice. Box plots show the median (center), 25th and 75th percentiles (box), and min to max (whiskers). Source data

Astrocyte maturation is influenced by crosstalk with neurons and other cell types in their local environment^54–61^. To ascertain which ligands and receptors participate in neuron–astrocyte interactions in each septal nucleus, we performed CellPhoneDB^62^ on defined neuron and astrocyte populations from both the MS and the LS (Fig. 4a and Extended Data Fig. 7a–d). Our data revealed that distinct ligand–receptor pairs were exclusively enriched in either MS or LS subsets of neurons and astrocytes (Fig. 4b and Extended Data Fig. 7e–g). Furthermore, we observed that several ligand–receptor pairs are not only region specific but also age dependent (Fig. 4c,d). Our previous research established that SHH, a ligand originating from deep-layer cortical neurons, binds to its receptor Ptch1 on cortical astrocytes. SHH signaling is necessary and sufficient to promote their layer-enriched molecular identity^58^. Our snRNA-seq and MERFISH analyses show that *Shh* expression is highly enriched in subsets of MS neurons (MSNs) (Fig. 4e,g and Extended Data Fig. 7e), where it increases over postnatal development (Fig. 4f). FGF family members exhibit enriched expression in LS neurons (LSNs) (Fig. 4e,h and Extended Data Fig. 7f,h), increasing with age (Fig. 4f), implying that these neuron-derived ligands could contribute to region-specific astrocyte diversification. Notably, SHH receptor Ptch1 and FGF receptor FGFR3 are known to be highly enriched in astrocytes throughout the CNS^58,63^. We examined the expression of genes previously shown to be downstream of SHH and FGF2 signaling in septal astrocytes^45,58^ and observed that many of these genes exhibit marked enrichment in the P21 MSAs and LSAs, respectively (Fig. 4i,k). *Lhx2*, a transcription factor downstream of FGF2 signaling^45^, is exclusively expressed in LSAs (Extended Data Fig. 7i,j). Downstream gene expression increased over time, mirroring the temporal increase of SHH and FGF ligand expression (Fig. 4j,l). To evaluate the role of SHH in regulating gene programs in astrocytes, we performed bulk RNA-seq on in vitro immunopanned astrocytes isolated from the whole brain after 3 d of treatment with the SHH agonist SAG (smoothened agonist)^64^ (Fig. 4m). Our analysis revealed that genes such as *Slc6a11* and *Ptch1* were significantly upregulated in experimental groups (Fig. 4n). To further validate whether *Slc6a11* expression is driven by SHH signaling pathway activation in vivo, we used an astrocyte-specific conditional mutant mouse model, wherein SHH signaling is hyperactivated by the conditional ablation of Ptch1 (Ptch1cKO)^58,65^. We observed that *Slc6a11* transcripts in LS were significantly increased in tdT^+^ astrocytes of juvenile and adult Ptch1cKO mice compared with wild-type (WT; Fig. 4o,p and Supplementary Fig. 5a,b). However, we did not observe a significant increase in MSAs despite the loss of *Ptch1*, which could be attributed to saturating levels of SHH signaling already present in the MS. This suggests that activation of SHH signaling in astrocytes is sufficient to drive *Slc6a11* expression in both the developing and the adult septum. To further test whether FGF signaling acts as an LSN-derived cue contributing to the molecular identity of LSAs, we performed RNA-seq on in vitro astrocytes exposed to recombinant FGF2 and FGF10 proteins for a week. However, we did not observe a significant number of DEGs in experimental groups compared with controls (Supplementary Fig. 5c–e); this could be owing to the possible requirement for additional conditions, such as the cell–cell interactions facilitated by three-dimensional cultures, to stimulate FGF signaling in astrocytes in vitro, illustrated in previous studies^45^. Collectively, our findings demonstrate that MSAs and LSAs undergo regional subtype-specific signaling crosstalk that is exclusive to their local environments. Based on their neuronal expression and target gene enrichment, SHH and FGF may function as two such neuron-derived cues that play important roles in shaping the specialized molecular identities of MSAs and LSAs, respectively.

### Transplanted astrocytes adopt local cell features

**Fig. 5:**
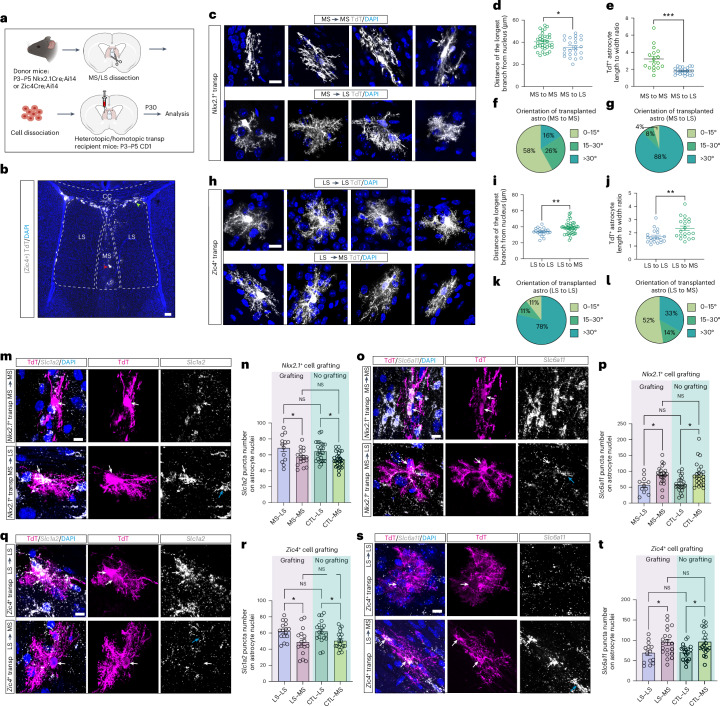
Transplanted astrocytes acquire local astrocyte features. **a**, Experimental design for cell transplantation (transp) experiments in the septum. **b**, Representative image of *Zic4*-derived cells at P30, after being grafted into the MS at P4. Immunostaining for tdTomato and DAPI. The red and green arrows indicate grafted cells in MS and LS, respectively. Scale bar, 100 µm. **c**,**h**, Immunostaining for tdTomato and DAPI in grafted *Nkx2.1*-derived (**c**) and *Zic4*-derived (**h**) astrocytes in heterotopic and homotopic conditions. Scale bars, 10 µm. **d**,**e**, Quantification of the length of the longest branch (**d**) and the length to width ratio (**e**) in heterotopic and homotopic conditions (*n* = 5–9 transplantations, 17–35 astrocytes analyzed for each condition). **f**,**g**, Pie charts showing the percentage of *Nkx2.1*-grafted astrocytes displaying the indicated angle ranges in homotopic (**f**) and heterotopic (**g**) conditions (*n* = 5–6 transplantations, 19–24 astrocytes analyzed for each condition). **i**,**j**, Quantification of the length of the longest branch (**i**) and the length to width ratio (**j**) in heterotopic and homotopic conditions (*n* = 5 transplantations, 19–40 astrocytes analyzed for each condition). **k**,**l**, Pie charts showing the percentage of *Zic4*-grafted astrocytes displaying the indicated angle ranges in homotopic (**k**) and heterotopic (**l**) conditions (*n* = 5 transplantations, 19–21 astrocytes analyzed for each condition). **m**,**o**, In situ hybridization of *Slc1a2* (**m**) and *Slc6a11* (**o**) combined with tdTomato staining in transplanted *Nkx2.1*-derived astrocytes. The white arrows indicate transplanted astrocytes and the blue arrow a host astrocyte. Scale bars, 10 µm. **n**,**p**, Quantification of *Slc1a2* (**n**) and *Slc6a11* (**p**) transcripts on astrocyte nuclei in both *Nkx2.1*-grafted and nongrafted groups (one-way ANOVA and Tukey’s multiple-comparison test; *n* = 4–6 transplantations, 11–26 astrocytes analyzed for each group). **q**,**s**, In situ hybridization of *Slc1a2* (**q**) and *Slc6a11* (**s**) combined with tdTomato staining in transplanted *Zic4*-derived astrocytes. Scale bars, 10 µm. **r**,**t**, Quantification of *Slc1a2* (**r**) and *Slc6a11* (**t**) transcripts on astrocyte nuclei in both *Zic4*-grafted and nongrafted groups (one-way ANOVA and Tukey’s multiple-comparison test, *n* = 4–6 transplantations, 14–20 astrocytes analyzed for each group). Data are presented as the mean ± s.e.m. *P < 0.05, **P < 0.01, ***P < 0.001. When *P* > 0.05, it is NS. Source data

**Extended Data Fig. 8:**
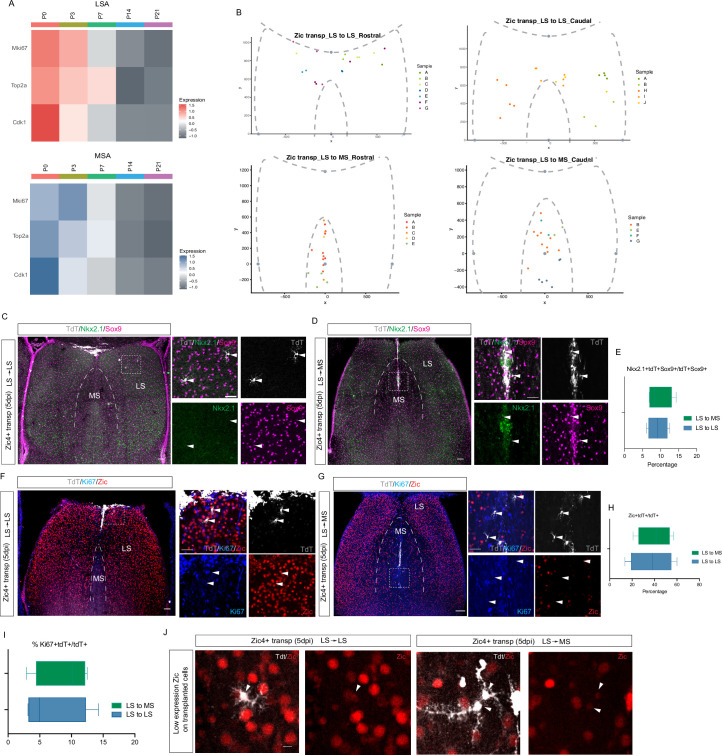
Proliferation capacity, location and the lineage-related gene expression patterns of grafted cells. **a**) Heatmap analysis of snRNAseq data indicating cell cycle genes enriched in the first few days after birth, including *Mki67*, *Top2a* and *Cdk1*. **b**) Location of Zic4-transplanted astrocytes in the septum at P30. Different colors indicate different samples. The four grey dots in each plot represent septal region indicators: the left grey dot indicates the left wall edge of the septum, the right grey dot indicates the right wall edge of the septum, the grey dot in the MS indicates the center of the MS, the top grey dot marks the midline. These grey dots were averaged from different samples used for analysis. N = 7-10 transplants, a total of 39-40 cells were analyzed. **c**, **d**) Immunostaining for tdTomato, Nkx2.1 and Sox9 in homotopic (**c**) and heterotopic (**d**) Zic4-transplanted samples 5 days post-transplantation. White boxes indicate high magnification images shown in the right panels. White arrows indicate transplanted cells positive for tdTomato and Sox9, but negative for Nkx2.1. Scale bar: 100 µm (left), 50 µm (right). **e**) Quantification of the percentage of Nkx2.1-postive transplanted astrocytes in the entire astrocyte population 5-day post-transplantation, LS to MS (9.44%) and LS to LS (9.22%), N = 4 transplants per each condition. **f**, **g**) Immunostaining for tdTomato, Ki67 and Zic in homotopic (**f**) and heterotopic (**g**) Zic4-transplanted samples 5 days post-transplantation. White boxes indicate high magnification images shown in the right panels. White arrows indicate transplanted cells positive for tdTomato, but negative for both Ki67 and Zic. Scale bar: 100 µm (left), 50 µm (right). **h**) Quantification of Zic-positive transplanted cells in the transplanted population 5-day post-transplantation, LS to MS (40.9%) and LS to LS (37.5%), N = 4 transplants for each condition, **i**) Quantification of the percentage of Ki67-positive transplanted cells in the entire transplanted population, LS to MS (8.88%) and LS to LS (6.80%), N = 4 transplants. **j**) Representative images showing low Zic expression in transplanted cells in both conditions. White arrows indicate grafted cells with low Zic expression. Scale bar: 10 µm. Box plots in E, H and I show the median (center), 25th and 75th percentiles (box), and min to max (whiskers). Source data

**Extended Data Fig. 9:**
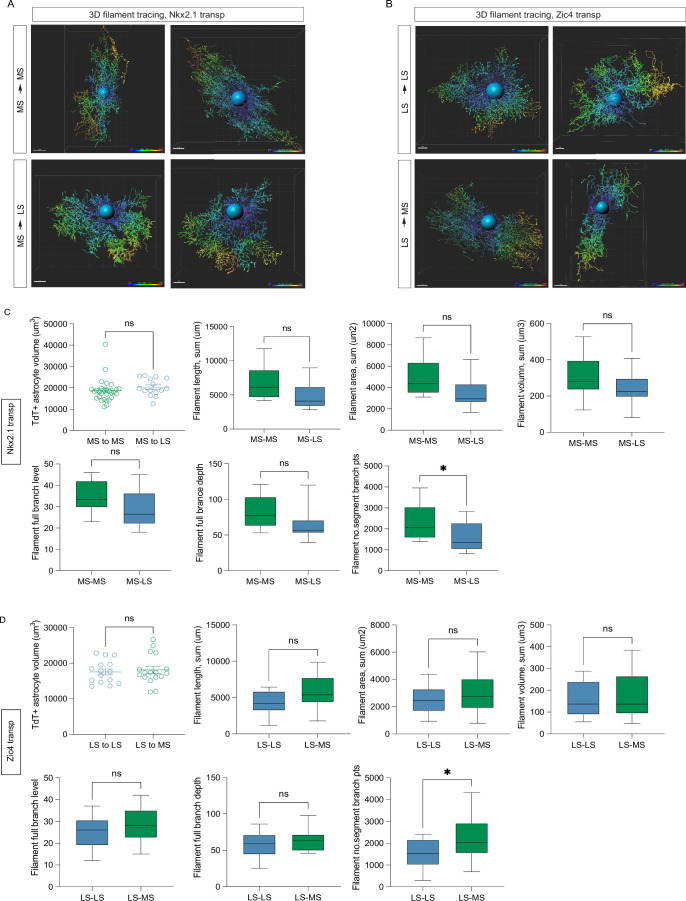
Analysis of morphological complexity in transplanted astrocytes. **a**) Representative filament tracing of Nkx2.1-grafted astrocyte branches within the MS and LS. **b**) Representative filament tracing of Zic4-grafted astrocyte branches within the LS and MS. **c**) Filament analysis of Nkx2.1-grafted astrocyte branches within the MS and LS (two-tailed, unpaired t-test with Welch’s correction, N = 4-5 transplants, a total of 10-29 astrocytes were used for filament tracing and analysis). **d**) Filament analysis of Zic4-grafted astrocyte branches within the LS and MS (two-tailed, unpaired t-test with Welch’s correction, N = 5-8 transplants, a total of 13-17 astrocytes were used for filament tracing and analysis). Dot graphs in C, D show the mean ± s.e.m. Box plots in C and D show the median (center), 25th and 75th percentiles (box), and min to max (whiskers). *p < 0.05, **p < 0.01, ***p < 0.001, ****p < 0.0001. When the p-value was greater than 0.05, it was stated as non-significant (ns). Source data

**Extended Data Fig. 10:**
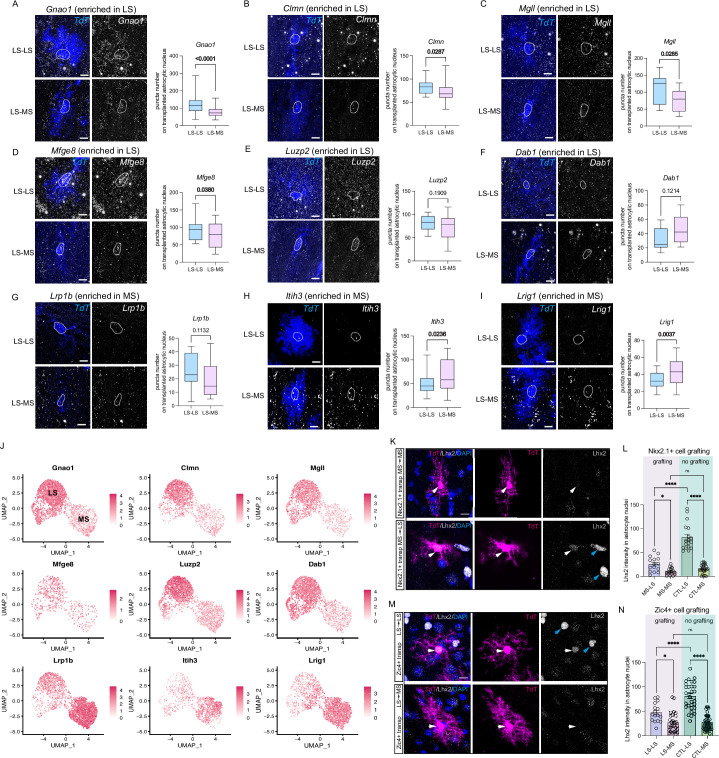
Grafted astrocytes adopt local astrocyte molecular features. **a-i**): HCR RNA-FISH/RNAscope analysis of genes enriched in LS and MS astrocytes on P30 transplanted tdT+ astrocytes: *Gnao1* (**a**), *Clmn* (**b**), *Mgll* (**c**), *Mfge8* (**d**), *Luzp2* (**e**), *Dab1* (**f**), *Lrp1b* (**g**), *Itih3* (**h**) and *Lrig1* (**i**). Dash circles indicate astrocyte nuclei. Scale bar: 10 µm. Statistics test: two-tailed, unpaired t-test with Welch’s correction. N = 5 transplants per each condition. **j**) Feature plots of genes enriched in P21 LS or MS astrocytes selected for HCR analysis. **k**) Immunostaining for Lhx2 (grey), tdTomato (magenta) and DAPI (blue) in transplanted *Nkx2.1*-derived astrocytes. White arrows indicate transplanted astrocytes; blue arrows indicate host astrocytes. Scale bar: 10 µm. **l**) Quantification of Lhx2 intensity on astrocytes nuclei on both grafting and no grafting groups (One-way ANOVA, Tukey’s multiple comparisons test, N = 4-6 transplants, 15-25 astrocytes were analyzed for each group). **m**) Immunostaining for Lhx2 (grey), tdTomato (magenta) and DAPI (blue) in P30 transplanted Zic4-derived astrocytes. White arrows indicate transplanted astrocytes and blue arrows indicate host astrocytes. Scale bar: 10 µm. **n**) Quantification of Lhx2 intensity on astrocyte nuclei on both grafting and no grafting groups (One-way ANOVA, Tukey’s multiple comparisons test, N = 4-6 transplants, 16-40 astrocytes were analyzed for each group). Box plots in A-I show the median (center), 25th and 75th percentiles (box), and min to max (whiskers). Bar graphs in L, N show the mean ± s.e.m. Source data

Astrocyte molecular identities are thought to arise through a combination of developmentally determined factors related to their lineage origins and extrinsic cues from their local environment^22,23^. However, the relative contribution of intrinsic programming versus environmental factors has not been fully determined. To address these questions, we performed homotopic and heterotopic transplantations of fluorescently labeled *Nkx2.1*-derived MS and *Zic4*-derived LS cells of P3–P5 mice into the septal region of WT mice of the same age (Fig. 5a). *Ki67* staining of *Zic4*-derived astrocytes transplanted into either the LS or the MS revealed a low number of cycling transplanted cells 5 d after transplantation (Extended Data Fig. 8a,f,g,i), suggesting that these cells exit the cell cycle and follow similar developmental trajectories in both regions. We examined the morphology and gene expression of transplanted cells at P30, a time point at which most cells had differentiated into astrocytes and oligodendrocytes, as neuronal survival throughout the procedure was limited (Fig. 5b). Heterotopically transplanted *Nkx2.1*-derived astrocytes within the LS exhibited a shorter and more angled branch pattern compared with homotopically transplanted controls, and more closely resembled local endogenous LSAs (Fig. 5c–g and Extended Data Fig. 9a,c). Similarly, *Zic4*-derived astrocytes grafted into the MS displayed elongated and less angled branches compared with controls (Fig. 5h–l and Extended Data Figs. 8b and 9b,d). Using immunostaining in *Zic4*-derived cells 5 d post-transplantation, we observed very low *Nkx2.1* and *Zic* levels in both homotopic and heterotopic *Zic4*-derived astrocytes (Extended Data Fig. 8c–e,j), consistent with the expression profile of the endogenous population (Extended Data Fig. 1i–k). To examine whether transplanted astrocytes acquire their mature molecular identities, we probed the expression of *Slc1a2* and *Slc6a11* transcripts, normally enriched in late-stage LSAs and MSAs, respectively (Fig. 2n,o). *Nkx2.1*-derived astrocytes transplanted into the LS exhibited an upregulation of *Slc1a2* transcripts and a downregulation of *Slc6a11* transcripts (Fig. 5m–p). Conversely, *Slc1a2* transcripts were decreased whereas *Slc6a11* transcripts were increased in *Zic4*-derived astrocytes transplanted into the MS when compared with homotopic transplants (Fig. 5q–t). Using our MERFISH data and scRFE analysis^66^ (Supplementary Table 2), we identified a set of genes that are necessary and sufficient to classify MSA and LSA clusters. We then performed RNAscope and multiplexed, quantitative, high-resolution RNA-FISH (HCR RNA-FISH)^67^ to assess the expression of those genes. We found that the expression of most LS-enriched genes, including *Gnao1*, *Clmn*, *Mgll* and *Mfge8*, were downregulated in *Zic4*-derived astrocytes within the MS, whereas the expression of MS-enriched genes, including *Itih3* and *Lrig1*, was upregulated in these transplanted cells (Extended Data Fig. 10a–j). In addition, we found that Lhx2 levels were increased in *Nkx2.1*-derived astrocytes transplanted into the LS and diminished in *Zic4*-derived astrocytes within the MS (Extended Data Fig. 10k–n). However, these expression patterns did not perfectly mirror those of the endogenous local astrocytes, because homotopically transplanted LSA had reduced Lhx2 compared with resident astrocytes, suggesting that this could be due to the transplantation procedure itself. Taken together, our findings suggest that extrinsic cues play a pivotal role in shaping the ultimate molecular and morphological identities of septal astrocytes, such that cells from a different lineage origin can still adopt functional features consistent with the local environment.

## Discussion

Here we have shown that astrocytes allocated to the MS and LS have distinctive progenitor origins, molecular profiles and morphologies suited to their specialized roles within each subregion. Our genetic fate mapping revealed that MSAs are derived from *Nkx2.1*-expressing progenitors, likely located in the MGE and/or POA^5,12,28^, whereas LSAs are produced within the septal proliferative zone by progenitors that express *Zic* family transcription factors. However, the specific contribution of intrinsic or extrinsic mechanisms to astrocyte specification is not understood. The sharp segregation of *Nkx2.1*- versus *Zic4*-derived astrocytes might suggest that many of the distinctive features of MSAs and LSAs are genetically encoded through their lineages. However, our data support a model where the local cellular environment substantially contributes to the final specification of astrocytes. Our transplantation experiments show that extrinsic factors are sufficient to induce astrocytes to adopt both the morphological and the molecular features of their local environment. Nevertheless, intrinsically encoded factors related to lineage origins likely play important roles in early developmental events such as the migration and positioning of astrocyte precursors into the LS or MS. This idea is strongly supported by the decreasing levels of *Nkx2.1* and *Zic* in both postnatal and transplanted astrocytes, which suggest that the role of these transcription factors diminishes as astrocytes mature. Once cells reach their final positions, local factors appear to take over, as the complement and concentration of extrinsic signaling cues dictate the final specification of astrocytes. This type of mechanism fits with the overall view of astrocytes as sensors of local environmental conditions, with the capacity to adjust to them to maintain homeostasis and support circuit adaptations. This model aligns with the cellular and molecular plasticity that we observed in our heterotopic transplantations. In the future, it will be important to determine the constraints on astrocyte plasticity that may be dictated by lineage origins or other factors and whether there is a critical developmental window during which astrocytes can adopt region-specific features. This will have important implications for the utilization of astrocytes in cell-based therapies.

The complex morphologies of astrocytes are essential for the performance of their diverse functions in the brain, allowing them to interact with a range of cell types, including neurons, other glia and blood vessels. MSAs exhibit elongated branches that align parallel to axons, like the fibrous astrocytes found in white matter regions^29,68^. This alignment suggests a potential influence of the dense fibers that traverse the MS in shaping the morphology of adjacent astrocytes. However, the specific factors originating from the local environment that contribute to this distinctive astrocyte morphology remain largely unidentified. Recent research efforts have explored the gene networks related to astrocyte morphology across a diverse range of brain regions and disease models^47^. Their findings suggest a complex interplay between the anatomical structure of local neurons and astrocyte-autonomous molecules in shaping astrocyte morphology. Future research should address the precise molecular interactions governing astrocyte morphogenesis within the septum, providing insight into both the diverse forms of astrocytes within this specific region and the broader spectrum of astrocyte morphologies and their alterations in disease.

Our snRNA-seq data reveal that MSAs and LSAs exhibit lineage-specific molecular identities during development and highlight two critical periods of astrocyte molecular specification: P0–P3, a phase marked by axonogenesis and neuron wiring, and P7–P14, a peak period of synapse formation, assembly and pruning^69^. Our data show that astrocyte–neuron interactions are not only age dependent, but also region and subgroup specific, with corresponding ligands and receptors enriched in subsets of MSNs and LSNs. These observations suggest the presence of unique interactions within specific subsets of neurons and astrocytes in the septum. SHH, a morphogen derived from neurons, signals to astrocytes expressing Ptch1, thereby regulating their molecular profiles in the cerebellum and cortex^24,58,70^. Here we show that the SHH signaling pathway strongly impacts septal astrocyte identity, highlighting the functional conservation of this ligand–receptor pair across different brain regions. It is interesting that FGF signaling does not appear to affect the molecular identity of in vitro astrocytes. This could mean that LSA gene profiles are not solely determined by a single signaling pathway, but rather a combination of signals and/or they are shaped by both local cues and physical interactions with neurons or other cell types, as shown in previous studies^45^. Moreover, our data unveiled several other critical ligand–receptor pairs that are likely to play a role in astrocyte specification, underscoring the extensive array of molecules involved in neuron–astrocyte interactions. These findings raise intriguing questions about the cooperative functional roles of these ligand–receptor pairs, especially in the context of age, cellular types and specific brain regions where these interactions take place.

Medial and lateral nuclei of the septum are distinguished by their patterns of connectivity with the hippocampus, where neurons in the MS project extensively to the hippocampus and receive inputs from a subset of GABA-ergic neurons in the same region^50,71^, whereas LSNs do not project directly to the hippocampus but receive substantial glutamatergic inputs from the CA region neurons^35,36,72^. These circuitry differences are reflected in the molecular profiles of MSAs and LSAs, whereby MSAs are highly enriched for GABA transporter genes (*Slc6a11* and *Slc6a1*) and LSAs show enrichment for glutamate transporter genes (*Slc1a2*, *Slc1a3* and *Slc1a4*). There is evidence that glutamate and GABA release induce transcriptional, cellular and functional changes in astrocytes^46,73,74^. However, whether glutamatergic and GABA-ergic neurotransmitter release is entirely responsible for transporter specialization is not entirely clear. Our data suggest that neuron-derived factors such as SHH and FGF may play important roles in astrocyte functional specialization in the septum. We show that astrocyte-specific activation of SHH signaling is sufficient to promote the upregulation of *Slc6a11* in LSAs. We also observed increased expression of *Slc1a2* and *Lhx2*, downstream targets of FGF signaling, in heterotopically transplanted LSAs^45^. These findings support our view that neuron-derived cues are essential for the expression of functional genes that specialize astrocytes for their circuit-specific roles.

Collectively, our data define the molecular heterogeneity of astrocytes within the septum and uncover the mechanisms that underlie astrocyte specification during septal development. Recent technological advancements have revealed new functions of astrocytes in both health and disease, shedding light on their potential as therapeutic targets. Understanding the intricate interactions between astrocytes and other cell types during development may provide insights into astrocyte regulation during circuit formation and refinement, thereby inspiring new ideas for therapeutic interventions^75–77^.

## Methods

### Experimental animals

All animal procedures conducted in this study followed experimental protocols approved by the Institutional Animal Care and Use Committee of Harvard Medical School (HMS; no. IS00000677-3) and the University of California, San Francisco (UCSF; nos. AN191974 and AN205156-00B). Mouse housing and husbandry conditions were performed in accordance with the standards of the Center of Comparative Medicine at HMS and the Laboratory Animal Resource Center (LARC) at UCSF. Embryonic (E) day 14, E17 and postnatal (P) days 1–35 mice were used for the present study. The embryonic and postnatal mouse ages for each experiment are indicated in figures and figure legends. Mouse lines: CD1 (strain code 022, Charles River Laboratories); the following are all from the Jackson Laboratory: C57BL/6J-Tg(Nkx2-1-cre) 2Sand/J(Nkx2.1-Cre) (cat. no. 008661), B6;129-*Gt(ROSA)26Sor*^*tm5(CAG-Sun1/sfGFP)Nat*^/J (cat. no. 021039), B6.Cg-*Gt(ROSA)26Sor*^*tm14(CAG-tdTomato)Hze*^/J(Ai14) (cat. no. 007914), Tg(Slc1a3-cre/ERT)1Nat/J Glast-CreERT2 (cat. no. 012586y), B6N.129-*Ptch1*^*tm1Hahn*^/J(Ptch1^flox/flox^) (cat. no. 012457) and Tg(Aldh1l1-EGFP,-DTA)D8Rth/J (cat. no. 026033); and Zic4Cre (Kessaris lab, UCL^27^). No statistical methods were used to predetermine sample sizes but our sample sizes are similar to those reported in our previous publications^5,58,78^. Samples or organisms were allocated into experimental groups using a random allocation method.

### Tamoxifen administration

Tamoxifen (Sigma-Aldrich) was dissolved in corn oil at a concentration of 20 mg ml^−1^ at 37 °C and stored at 4 °C. For mice injected at P10 (WT: Glast-CreERT2;Ai14; Ptch1cKO: Glast-CreERT2;Ptch1flox/flox;Ai14), 10–15 µl of tamoxifen was injected intraperitoneally for 3 d consecutively to induce efficient recombination. For astrocyte morphology analysis, we injected a single dose of tamoxifen into Glast-CreERT2;Ai14 mice to sparsely label astrocytes.

### Immunohistochemistry

Postnatal animals were transcardially perfused with phosphate-buffered saline (PBS) followed by 4% paraformaldehyde (PFA) and their brains were dissected out and post-fixed in 4% PFA overnight at 4 °C. Brains were sliced into 50-µm sections on a vibratome (Leica Microsystems, cat no. VT1200S). Sections were prepared and placed in blocking solution (0.3% Triton (Amresco) and 10% goat serum in PBS) for 1–2 h at room temperature (RT), followed by primary antibodies overnight at 4 °C and secondary antibodies for 1 h at RT. DAPI (Invitrogen) was added to the secondary antibody solution. The sections were mounted using ProLong Gold Antifade Mountant (Invitrogen). Cryosections consisted of the following: brains were cryoprotected in 30% sucrose/PBS overnight at 4 °C after post-fixation. Brains were embedded in optimal cutting temperature (OCT) compound (Sakura), frozen on dry ice and stored at −80 °C. Samples were sectioned at 20 µm on a cryostat (CryoStar, Thermo Fisher Scientific, cat. no. NX70). Images were acquired using a Leica SP8 or STELLARIS laser point scanning confocal microscope; ×10, ×20, ×40, ×63 and ×100 objectives were used and images were further analyzed using Fiji. Brightness and contrast were adjusted as necessary for visualization, but the source images were kept unmodified. For astrocyte morphology analyses, *z*-stack images were collected to maximize the coverage of the entire astrocyte territory. The territory and the length from the nucleus to the end of the longest branch were measured in Fiji. For Imaris filament tracing, *z*-stack images of tdT^+^ astrocytes were acquired using a Leica Stellaris confocal and the FilamentTracer tool in Imaris 10.1 software was used to map individual astrocyte branches. Statistical data were automatically generated by software (for example, MATLAB, Prism 9). Primary or secondary antibodies included: Lhx2 (1:500–1,000, rabbit, Millipore, cat. no. ABE1402), red fluorescent protein (RFP; 1:500, rabbit, Rockland, cat. no. 600-401-379), Sox9 (1:500, goat, R&D, cat. no. AF3075), Olig2 (1:2,000, rabbit, Millipore, cat. no. AB9610), Zic (1:1,000, rabbit, gift from the Segal^79^ lab, DFCI), Nkx2.1 (1:500, clone-8G7G3/1, mouse, Santa Cruz, cat. no. 53136), GFP (1:500, chicken, Aves, cat. no. GFP-1020), RFP (1:500, chicken, Rockland, cat. no. 600-901-379), PSD95 (1:500, rabbit, Thermo Fisher Scientific, cat. no. 51-6900), VGluT1 (1:500, guinea-pig, Millipore, cat. no. AB5905), VGAT (1:500, clone-Gp117G4, guinea-pig, Synaptic Systems, cat. no. 131308), gephyrin (1:500, clone-RbmAb7a, rabbit, Synaptic Systems, cat. no. 147008), calbindin (1:1,000, rabbit, Swant, cat. no. SKU CB38a), parvalbumin (1:500, clone-PARV-19, mouse, Sigma-Aldrich, cat. no. SAB4200545), goat polyclonal anti-chicken Alexa Fluor-488 and -546 (1:1,000, Thermo Fisher Scientific, cat. nos. A11039 and A11040), goat polyclonal anti-guinea-pig Alexa Fluor-488 (1:1,000, Thermo Fisher Scientific, cat. no. A11073), goat polyclonal anti-mouse Alexa Fluor-488 and -647 (1:1,000, Thermo Fisher Scientific, cat. nos. A21236 and A11001), goat polyclonal anti-rabbit Alexa Fluor-488 and -647 (1:1,000, Thermo Fisher Scientific, cat. nos. A11034 and A21245), donkey polyclonal anti-goat Alexa Fluor-647 (1:1,000, Thermo Fisher Scientific, cat. no. A21447) and donkey polyclonal anti-mouse Alexa Fluor-488 (1:1,000, Thermo Fisher Scientific, cat. no. A21202).

### snRNA-seq and analysis

The septum was dissected out from Nkx2.1Cre;Sun1GFP mice. Nuclei were isolated from septal tissue. The number of biological replicates used was as follows: for P0 and P3, two pooled samples were used (each pool contained three mice; their sex was not determined); for P7, two males were used; for P14, one female and one male; and for P21, two females and two males. Both GFP^+^ and GFP^−^ nuclei were sorted and counted for cell density, followed by the 10x Genomics platform (Chromium next GEM single-cell 3ʹ-reagent kits v.3.1(dual index)) and sequencing using the NovaSeq 6000 sequencer. Sequence reads were processed and aligned to the mouse genome GRC38 using the 10x Genomics Cell Ranger 6.0.0. Nuclei expressing <200 genes, total RNA counts <1,000 and/or a percentage of mitochondrial reads >5% were excluded from the analysis (for astrocyte cluster analysis, mitochondrial reads >2% of total reads were excluded from the analysis). For snRNA-seq analysis at different ages, a total of 166,418 nuclei (P0: 35,066 nuclei; P3: 36,692 nuclei; P7: 29,034 nuclei; P14: 19,926 nuclei; P21: 45,700 nuclei; GFP^+^: 72,573 nuclei; and GFP^−^: 93,845 nuclei) were used for downstream analysis in R (v.4.2.3). Low read counts were normalized and dimensional reduction was performed by principal component analysis (PCA) (*n* = 50), followed by regressing out the percentage mitochondrial reads and number of genes per nucleus. Data from different developmental stages were integrated using the Seurat (v.5.1.0) anchor-based integration pipeline with reciprocal PCA, as recommended by the authors when integrating samples with potentially nonoverlapping populations. Cell types were determined by examining the expression of canonical marker genes. For differential expression analysis, a Seurat object was generated, followed by the FindMarkers() function. GO enrichment analysis was performed using the enrichGO() function (v.4.6.2). Cortical, hippocampal and striatal scRNA-seq data were obtained from a previously published dataset^47^ (accession no. GSE198027) and integrated with P21 snRNA-seq septal astrocytes and normalized for subsequent analyses. FGF2 downstream targets have been shown previously^45^. Genes that were both FGF2 downstream targets and highly enriched in LS astrocytes were selected in Fig. 4k. SHH downstream targets have been shown previously^58^. Genes that were both SHH downstream targets (cut-off *P* < 0.01) and highly enriched in MS astrocytes, plus known SHH signaling pathway members (Ptch1, Smo and Gli family members)^80^ were selected in Fig. 4i. Cell–cell interaction analysis between different cell populations was performed using CellPhoneDB v.3.0 and method = ‘statistical_analysis’^62^. The ligand–receptor pairs expressed in both MS and LS neurons and astrocytes were excluded for further analysis. Transcription factor–target regulatory network analysis was performed using the SCENIC method^51^. A genome search space between 500 bp and 10 kb around the transcription start sites was used. For WGCNA analysis, the R package WGCNA v.1.7.3 was applied^81–83^. For scRFE analysis, the package scRFE (v.1.5.6) was applied to perform recursive feature elimination (RFE), selecting the most important genes for distinguishing cell clusters^66^.

### MERFISH

The brains of two P35 CD1 male mice were collected. Then, 10-µm tissue sections were obtained along the rostrocaudal axis and adhered to the MERSCOPE slide. The samples were permeabilized in 70% ethanol overnight, followed by cell boundary staining. A 500-gene panel was generated based on enriched genes in each astrocyte and neuron cluster, with approximately 100 astrocyte genes and 400 neuron genes. The samples were then hybridized using the gene panel mix, followed by gel embedding and clearing steps. Images were processed using the MERSCOPE instrument and analysis computer, along with MERSCOPE visualizer software to streamline the acquisition of high-quality MERFISH data. Cells with a volume >2,000 µm^3^, those with <100 transcripts and those with DAPI scores <500 were excluded from the analysis. A total of 160,301 cells was used for downstream analysis, including 31,797 astrocytes. Further clustering analysis and differential gene expression analysis were performed in spyder (v.3.9.14). The packages include numpy (v.1.26.4), scanpy (v.1.10.1), SpatialDE (v,1.1.3), NaiveDE (v,1.2.0), anndata (v.0.10.7) and pandas (v.2.2.2).

### In situ hybridization

PFA-fixed brain sections were collected and preserved in a freezing buffer (250 ml buffer: 70 g of sucrose, 75 ml of ethylene glycol, fill to 250 ml with 0.1 M of sodium phosphate buffer). These brain sections were pretreated using the RNAscope fluorescent multiplex assay which involved using RNA probes specific to *Slc1a2*, *Slc6a11*, *Lrig1* and *Itih3*. For cell transplantation experiments, a multiplex RNAscope approach was employed by combining *Slc1a2*, *Slc6a11*, *Lrig1* and *Itih3* probes with tdTomato immunostaining to confirm the identity of the labeled cells as transplanted cells. *Nkx2.1* probe and GFP immunostaining were used to examine *Nkx2.1* expression patterns across developmental stages. Probes included (all from ACD): *Slc6a11* (cat. no. 492661-C3), *Slc1a2* (cat. no. 441341), *Lrig1* (cat. no. 310521), *Itih3* (cat. no. 840071) and *Nkx2.1* (cat. no. 434721).

### HCR RNA-FISH

The protocol in this work has been optimized^67,84^. The 50-µm-thick sections of PFA-fixed tissue were collected in freezing buffer. Slides were immersed in pre-chilled 4% PFA and fixed on ice for 30 min. After rinsing with PBS, slides underwent dehydration: 50% EtOH for 5 min, 70% EtOH for 5 min and 100% EtOH twice for 5 min each. Slides were then air-dried for 5 min at RT. Slides were incubated in proteinase K (10 µg ml^−1^) for 5 min at RT, followed by two PBS rinses. Next, slides were immersed in a pre-warmed probe hybridization buffer for 10 min at 37 °C, then incubated with probes (10 nM) overnight in a humidified chamber at 37 °C. The next day, slides were washed in a series of probe wash buffers and 5× SSCT (saline sodium citrate with Tween 20) mixtures at 37 °C for 15 min each: 75% probe wash buffer/25% SSCT, 50% probe wash buffer/50% 5× SSCT, 25% probe wash buffer/75% SSCT and, finally, 100% SSCT for 15 min at 37 °C, followed by a 5-min wash in 5× SSCT at RT. Slides were then incubated in pre-equilibrated amplification buffer for 30 min at RT, followed by overnight incubation with a snap-cooled hairpin mixture in a dark humidified chamber at RT. On the next day, excess hairpins were removed by washing in 5× SSCT at RT for 2× 30 min, followed by a 2× SSC (saline sodium citrate) rinse. DAPI (5 µg ml^−1^) was applied for 5 min and slides were mounted with Vectashield vibrance antifade mounting medium. Imaging was performed within 48 h of mounting. For the next round of probe hybridization, coverslips were removed by washing slides in 2× SSC. Slides were washed in 2× SSC for 2× 5 min at RT, followed by incubation with DNase I (250 U ml^−1^) for 1.5 h at RT in a humidified chamber. After washing with 2× SSC buffer for 6× 5 min, the pre-hybridization and imaging steps were repeated for the desired number of HCR rounds. The probes included (all from Molecular Instruments): *Gnao1* (cat. no. NM_001369049.1), *Clmn* (cat. no. NM_053155.2), *Mgll* (cat. no. NM_001166251.2), *Mfge8* (cat. no. NM_001045489.1), *Lrp1b* (cat. no. NM_053011.2), *Dab1* (cat. no. NM_001369049.1), *Luzp2* (cat. no. NM_178705.5) and *TdTomato* (cat. no. AAV52169).

### Immunopanning astrocyte culture

Astrocytes were purified using a previously published immunopanning protocol^85,86^. Petri dishes were first coated with species-specific secondary antibodies and then with an antibody against CD45 (BD, cat. no. 550539), a hybridoma supernatant against the O4 antigen^85^ and an antibody against HepaCAM (R&D Systems, cat. no. MAB4108), respectively. Whole mouse brains were dissected from P2 mice, with the meninges, olfactory bulbs and cerebellum removed. The remaining tissue was digested into a single-cell suspension using papain (Worthington, cat. no. LS 03126). The single-cell suspension was then sequentially passed through a series of 150-mm-diameter Petri dishes: two CD45-coated dishes to deplete microglia and macrophages, one O4-coated dish to deplete oligodendrocyte precursor cells and one HepaCAM-coated dish to select for astrocytes. After washing away nonadherent cells from the HepaCAM-coated dish with Dulbecco’s PBS, astrocytes were lifted using trypsin (Sigma-Aldrich, cat. no. T9935). The astrocytes were then seeded on poly(d-lysine)-coated plastic coverslips in a serum-free medium at a density of 75,000 cells per well on a 24-well plate. The serum-free medium contained 50% Neurobasal (Gibco, cat. no. 21103-049), 50% Dulbecco’s modified Eagle’s medium (Invitrogen, cat. no. 11960-044), 1× penicillin–streptomycin (Invitrogen, cat. no. 15140-122), 1 mM sodium pyruvate (Invitrogen, cat. no. 11360-070), 2 mM l-glutamine (Invitrogen, cat. no. 25030-081), 1× SATO^87^, 5 μg ml^−1^ of *N*-acetyl cysteine (Sigma-Aldrich, cat. no. A8199) and 5 ng ml^−1^ of heparin-binding epidermal growth factor-like growth factor (Sigma-Aldrich, cat. no. E4643). Half of the medium was replaced with fresh medium every 3 d. Drug treatment consisted of SAG (diluted in dimethyl sulfoxide (DMSO) to a concentration of 500 ng ml^−1^; Tocris, cat. no. 4366) and DMSO (control group) was added to 3-d in vitro (DIV) astrocyte cultures for 3 d. FGF2 (Peprotech, cat. no. 450-33) and FGF10 (R&D Systems, cat. no. 345-FG-025), both at a working concentration of 50 ng ml^−1^ (diluted in PBS), along with PBS as the control group, were added to in vitro astrocyte cultures (DIV2) for 7 d. The number of biological replicates used in bulk RNA-seq are as follows: DMSO control (3), SHH agonist SAG (3), PBS control (4), FGF2 (3) and FGF10 (3).

### RNA-seq analysis

In vitro astrocyte RNA was purified with an RNeasy Mini Kit (QIAGEN). The concentration and quality of the purified RNA were assessed with a BioAnalyzer (Agilent). The RNA was then reverse transcribed into complementary DNA and purified using the Lexogen QuantSeq 3ʹ-mRNA-Seq v.2 Library Prep Kit FWD with unique dual indices. Before the library amplification step, the PCR cycle number was determined using the PCR Add-on Reamplification Kit v.2 (Lexogen). Abundant BC1 transcripts, commonly found in mouse brain samples, were removed using Lexogen’s BC1 module during the RNA removal step. The library pools were quantified using a Qubit fluorometer and Agilent TapeStation 2200. Uniquely indexed libraries were pooled in equimolar ratios and sequenced on a NextSeq 2000 P3 (1.1 billion clusters) with index 1 and index 2 lengths of 12 bp each. On average, 59.41 million input reads were obtained per sample, with an average of 44.76 million uniquely mapped reads. Sequenced reads were aligned to reference genome assembly and gene counts were quantified using the STAR tool^88^ (v.2.7.10). Differential gene expression testing was performed by DESeq2 (ref. ^89^) (v.1.38.3). Significant genes were identified using a threshold: adjusted *P* < 0.01, log_2_(fold-change) > 1.

### Cell transplantation

P3–P5 CD1 mice were used as recipients and Zic4Cre;Ai14 or Nkx2.1Cre;Ai14 mice were used as donors. Brains were sliced by using a 0.5-mm brain matrix, and the MS and LS regions were dissected in Leibovitz L-15 medium (L15; Gibco). The tissue was digested into a single-cell suspension using papain. Dissociated cells were kept in ice-cold L15-containing DNase I (180 μg ml^−1^). Cells were then concentrated by spinning in a tabletop centrifuge for 5 min at 800*g*, followed by the removal of the supernatant. The final cell pellet was resuspended and mixed in a final volume of 1–6 μl of L15. This concentrated cell suspension was loaded into beveled glass micropipettes (~60–100 μm in diameter; Wiretrol 5 μl, Drummond Scientific Co.) prefilled with mineral oil and mounted on a microinjector as previously described^90^. The viability and concentration of the cells in the glass micropipette were determined using a hemocytometer, loaded with 100 nl of cells diluted 200× in 10 μl of L15 and 10 μl of Trypan Blue. Cells were injected into P3–P5 WT (CD1) mice. Before the injection of cells, the recipient mice were anesthetized by hypothermia (~3–5 min) and positioned in a clay head mold to stabilize the skull^91^. Micropipettes were positioned at an angle of 0° from vertical in a stereotactic injection apparatus and injected into the septum. The distance between the two eye corners was used to find the midline of the mouse brain. The injection coordinates to target the medial septum were 0 mm mediolateral (M/L) from the midline, 2.75 mm anteroposterior (A/P) from the eye corner and 3.2 mm dorsoventral (D/V) from the surface of the head. The coordinates to target the lateral septum were 0.5 mm M/L from the midline, 2.6 mm A/P from the eye corner and 3.0 mm D/V from the surface of the head. After the injections were completed, the transplanted mice were placed on a warming pad to recover from hypothermia and returned to their mothers afterwards. Brains were collected at P30. For morphology analysis, *z*-stack images were collected, maximizing the branch length of astrocytes using maximum intensity projection in ImageJ.

### Analysis of colocalized synaptic puncta

P21 GlastCreER;Ai14 mice were collected and sections were stained with antibodies against presynaptic and postsynaptic makers: VGluT1 and PSD95, or GAT and gephyrin. High magnification (×100, 1.44 numerical aperture objective plus ×1.5 optical zoom) *z*-stack images were obtained with a Leica SP8 confocal microscope. Each image includes individual astrocytes with 5-µm-thick *z*-stacks. The number of colocalized synaptic puncta on tdTomato^+^ astrocyte territories was analyzed using MATLAB^58^.

### Statistics and reproducibility

Cell counting was analyzed manually with ImageJ/Fiji cell counter (National Institutes of Health (NIH), v.2.0.0-rc-69/1,52p). Analysis of cell density was done in ImageJ by dividing the septum into its medial and lateral nuclei based on DAPI staining and the LS was divided into three subdivisions from dorsal to ventral. The area size of the region of interest was measured and the cells in these regions were counted. Quantification of synapse puncta in astrocyte territories and quantification of RNA puncta per area were carried out automatically using a data-processing pipeline (see above) in MATLAB R2019b. All statistical tests were performed in GraphPad Prism 9. For each experiment, the number of replicates (*n*) is indicated in the figure legends. The accepted value for significance was *P* < 0.05. Numbers of mice are reported in the figure legends. The experiments reported here were repeated independently at least three times, using mice from at least three different generations. No statistical methods were used to predetermine sample size for single experiments. Data distribution was assumed to be normal, but this was not formally tested. All samples were pooled to perform multiplexed snRNA-seq/MERFISH/bulk RNA-seq, so that different groups were processed in parallel, and no human judgment was involved. For other experiments, sample collection was not fully performed blind to the experimental conditions; however, the data analyses were performed either using automated data analysis procedures without consideration of experimental groups or the experimenters were fully blind to the experimental conditions. Data analysis was performed using the same parameters across groups. All data were used for analysis unless there were apparent failures of the experiment.

### Reporting summary

Further information on research design is available in the Nature Portfolio Reporting Summary linked to this article.

## Online content

Any methods, additional references, Nature Portfolio reporting summaries, source data, extended data, supplementary information, acknowledgements, peer review information; details of author contributions and competing interests; and statements of data and code availability are available at 10.1038/s41593-025-02007-z.

## Supplementary information

Supplementary Information Supplementary Figs. 1–5 and legends.

Reporting Summary

Supplementary Table 1 Supplementary Table 1, related to Fig. 3 SCENIC analysis revealing the transcription factor–target regulatory networks in septal astrocytes. ‘Genes’ indicates the list of target genes of each transcription factor. ‘Weights’ indicates the close linkage between each target and the corresponding transcription factor.

Supplementary Table 2 Supplementary Table 2, related to Fig. 5 The scRFE analysis identifying the significant genes defining P21 astrocyte clusters. Blue and red colors indicate the top 25 genes significantly associated with P21 MSA and LSA clusters, respectively. Two asterisks (^**^) denote genes present in both MSAs and LSAs. A higher Gini score reflects greater significance of the genes in representing the defined cluster.

## Source data

Source Data Fig. 1 Statistical source data.

Source Data Fig. 4 Statistical source data.

Source Data Fig. 5 Statistical source data.

Source Data Extended Data Fig. 1 Statistical source data.

Source Data Extended Data Fig. 2 Statistical source data.

Source Data Extended Data Fig. 7 Statistical source data.

Source Data Extended Data Fig. 8 Statistical source data.

Source Data Extended Data Fig. 9 Statistical source data.

Source Data Extended Data Fig. 10 Statistical source data.

## Data Availability

The snRNA-seq data, MERFISH and RNA-seq data have been deposited at the Gene Expression Omnibus (GEO) and are publicly available as of the date of publication. GEO accession nos. are listed as: snRNA-seq data (GSE281738), MERFISH (GSE282127) and bulk RNA-seq data (GSE281480). Source data are provided with this paper.

## Code availability

No original code was used in this study. The code and pipelines used in this study can be provided upon reasonable request.
