# Supplementary material for: Astrocyte specification in the mouse septum is shaped by both developmental origin and local signals
Source: Nat Neurosci. 2025 Jul 28;28(8):1676–87. doi: 10.1038/s41593-025-02007-z (PMC12321577; doi:10.1038/s41593-025-02007-z)
Supplement: Supplementary file 1 — Supplementary Figs. 1–5 and legends. [file 41593_2025_2007_MOESM1_ESM.pdf]

nature neuroscience

Supplementary information https://doi.org/10.1038/s41593-025-02007-z

Astrocyte specification in the mouse septum is shaped by both developmental origin and local signals

In the format provided by the authors and unedited

Supplementary Fig.1

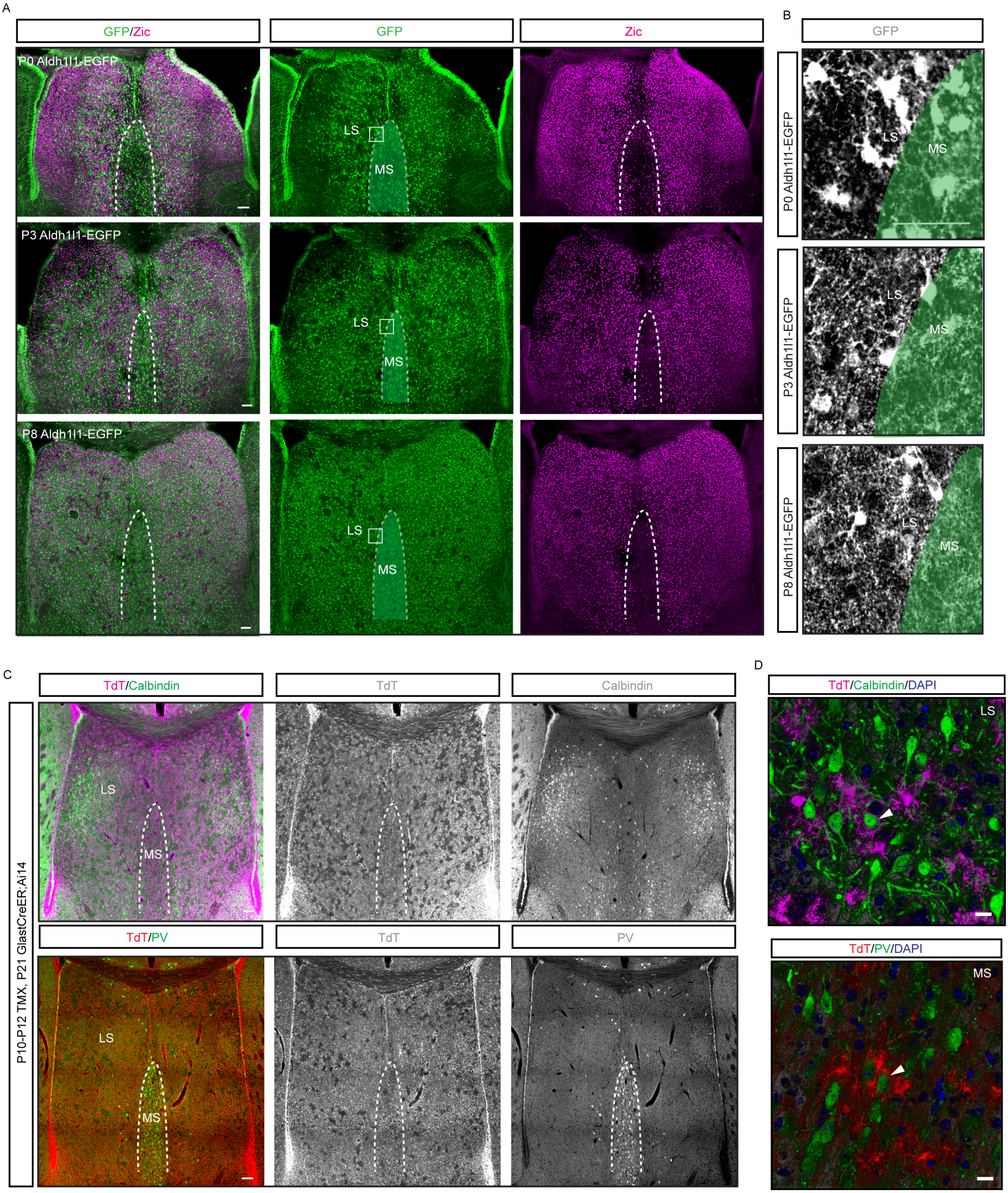

Supplementary Fig.2

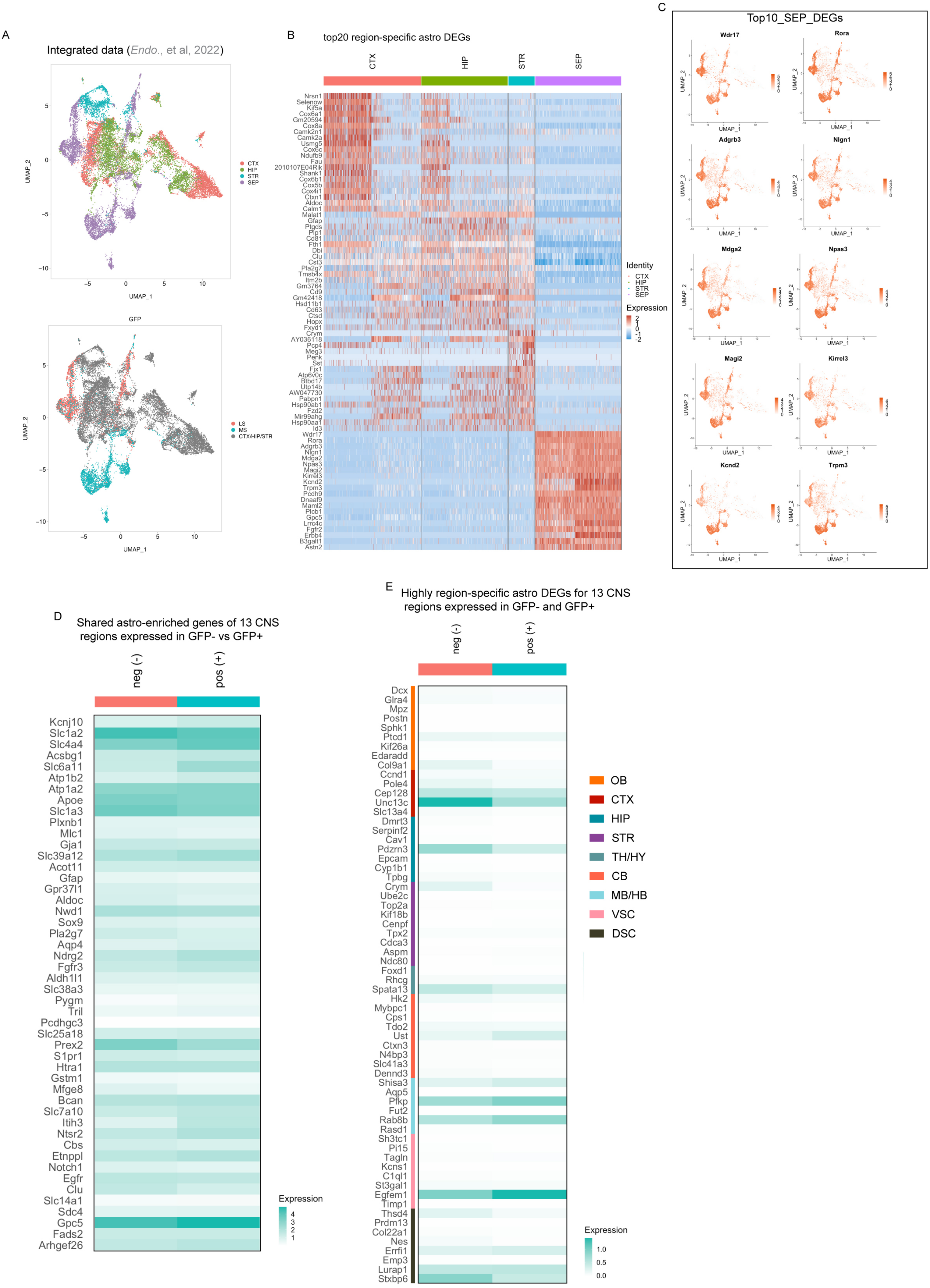

Supplementary Fig.3

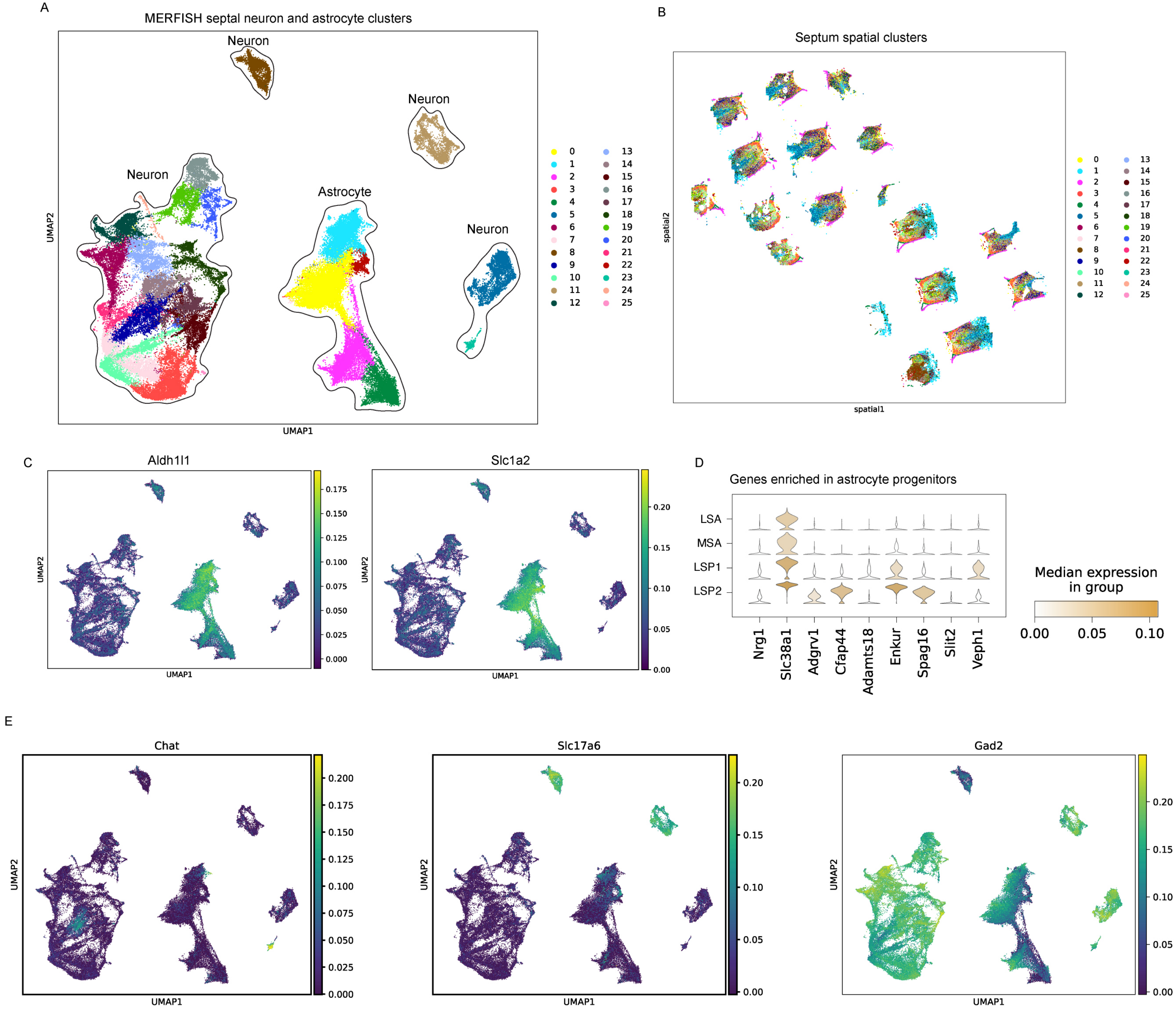

Supplementary Fig.4

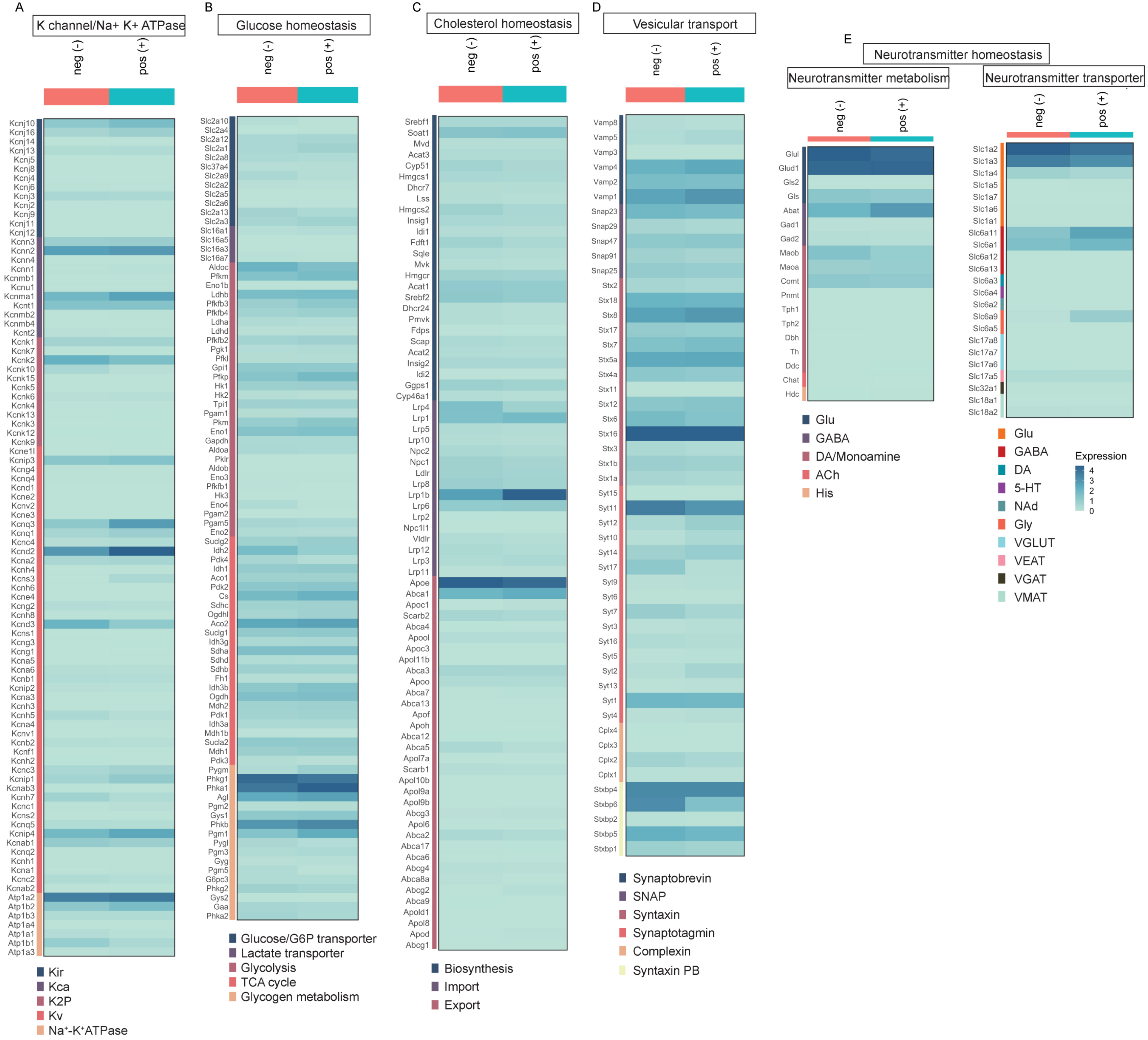

Supplementary Fig.5

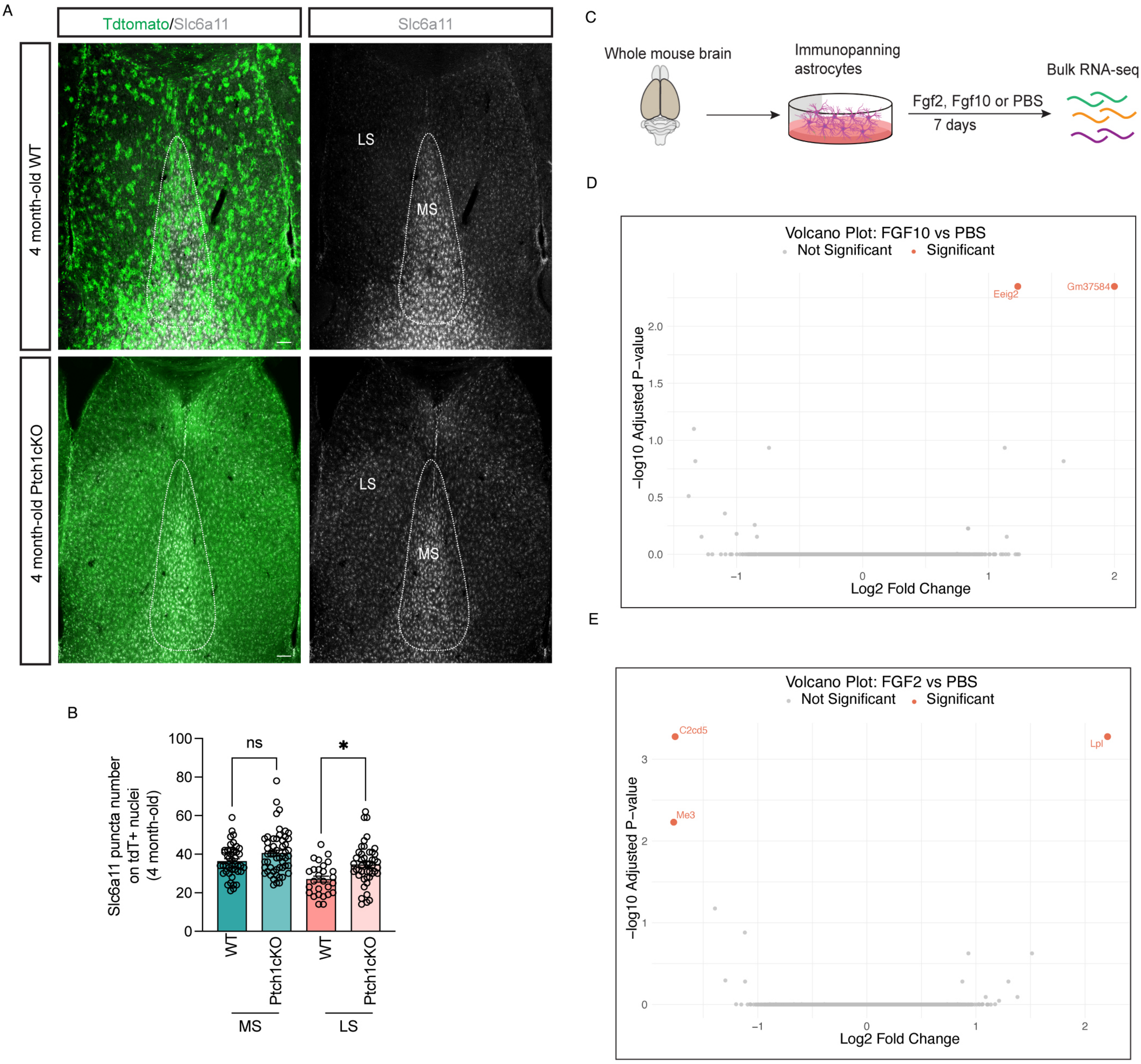

Supplementary Fig.1, related to Fig.1: Astrocyte cellular properties in the septum. **A**, **B**) Immunostaining for GFP in P0, P3, and P8 Aldh1l1-EGFP mice; MS and LS boundaries were distinguished by Zic staining (LS marker) (**A**). White boxes indicate the regions selected for high magnification images (right panel) (**B**). Scale bars: left: 100 µm, right: 10 µm. **C**) Immunostaining for tdTomato (magenta), Calbindin, and Parvalbumin in tamoxifen-induced P21 GlastCreER; Ai14 mice (tamoxifen injected at P10-P12). Scale bar: 100 µm. **D**) High magnification images showing Calbindin+ and PV+ neurons closely ensheathed by adjacent tdTomato+ astrocytes in the LS and MS respectively. Scale bar: 10 µm.

Supplementary Fig.2, related to Fig.2: Septal astrocytes exhibit region-specific molecular signatures. **A**) UMAP plot showing astrocyte clusters in four distinct regions (top) and segregation between MS astrocytes and other regions based on GFP expression (bottom). CTX = cortex, HIP = hippocampus, STR = striatum, SEP = septum. **B**) Heatmap showing the top 20 region-specific enriched genes in the CTX, HIP, STR and SEP. **C**) Feature plots of the top 10 septal DEGs. **D**) Heatmap showing genes shared across the 13 CNS regions indicated in panel E, as expressed in P21 GFP+ and GFP- septal astrocytes. **E**) Heatmap plot showing the enrichment of region-specific DEGs from 13 CNS regions (olfactory bulb (OB), motor cortex (MCX), somatosensory cortex (SCX), visual cortex (VCX), hippocampus (HIP), striatum (STR), thalamus (TH), hypothalamus (HY), cerebellum (CB), midbrain (MB), hindbrain (HB), ventral spinal cord (VSC) and dorsal spinal cord (DSC)) in GFP- and GFP+ septal astrocytes.

Supplementary Fig.3, related to Fig.2: MERFISH analysis of P35 septal cells. A) UMAP plot of MERFISH data revealing 25 subclusters of P35 septal astrocytes and neurons. **B**) Spatial transcriptomics analysis showing 25 subcluster locations in the septum along the rostro-caudal axis. **C**) Astrocyte clusters in MERFISH were identified by general astrocyte markers: *Aldh1l1* and *Slc1a2*. **D**) Genes enriched in LSP cluster (snRNAseq, **Extended Data Fig. 3I**) show high enrichment in MERFISH clusters LSP1

and LSP2. **E**) Neuron clusters in MERFISH were identified by general neuron markers: *Chat, Slc17a6, Gad2*.

Supplementary Fig.4, related to Fig.3: Expression of genes regulating astrocytic core function in septal astrocytes. A-E) Heatmaps showing the average expression of genes for potassium channels and Na+-K+ ATPases (**A**), glucose homeostasis (**B**), cholesterol homeostasis (**C**), vesicular transport (**D**), and neurotransmitter homeostasis (**E**) in P21 GFP+ vs GFP- astrocytes. Kir: inwardly rectifying K+ channel, Kca: calcium-activated K+ channel, K2P: two-pore domain K+ channel, Kv: voltage-gated K+ channel, Glu: glutamate, DA: dopamine, Ach: acetylcholine, 5-HT: serotonin, His: histamine, Ad: Adrenaline, Gly: glycine, VGLUT: glutamate vesicular transporter, VEAT: vesicular excitatory amino acid transporter, VGAT: vesicular GABA transporter, VMAT: vesicular monoamine transporter, VAChT: vesicular acetylcholine transporter.

Supplementary Fig.5, related to Fig.4: Shh and Fgf signaling regulate gene expression in septal astrocytes. A) *In situ* hybridization of *Slc6a11* combined with immunostaining for tdTomato in 4 month-old tamoxifen-induced GlastCreER;Ai14 (WT) and GlastCreER; Ptch1fl/fl; Ai14 (Ptch1cKO) mice. Tamoxifen was injected in 4 month-old mice, samples were collected after 3 weeks. Scale bar: 100 µm. **B**) Quantification of *Slc6a11* puncta number on tdT+ astrocyte nuclei (One-way ANOVA, Turkey's multiple comparisons test, N = 4 mice, 28-54 astrocytes in each condition). **C**) Schematic of the methods for bulk RNA-seq of immunopanned astrocytes under treatment with Fgf2 and Fgf10. **D**) Volcano plot showing differential gene expression in Fgf2 vs. PBS, and Fgf10 vs. PBS. N = 4 (PBS), 3 (Fgf2), 3 (Fgf10) replicates. *p < 0.05, **p < 0.01, ***p < 0.001, ****p < 0.0001. When the p-value was greater than 0.05, it was stated as non-significant (ns).
