# Supplementary material for: Astrocyte specification in the mouse septum is shaped by both developmental origin and local signals
Source: Nat Neurosci. 2025 Jul 28;28(8):1676–87. doi: 10.1038/s41593-025-02007-z (PMC12321577; doi:10.1038/s41593-025-02007-z)
Supplement: Supplementary file 2 — Reporting Summary [file 41593_2025_2007_MOESM2_ESM.pdf]

nature portfolio

Corresponding author(s): Corey C. Harwell

Last updated by author(s): Apr 25, 2025

Reporting Summary

Nature Portfolio wishes to improve the reproducibility of the work that we publish. This form provides structure for consistency and transparency in reporting. For further information on Nature Portfolio policies, see our Editorial Policies and the Editorial Policy Checklist.

Statistics

For all statistical analyses, confirm that the following items are present in the figure legend, table legend, main text, or Methods section.

n/a	Confirmed	
☐	☒	The exact sample size (*n*) for each experimental group/condition, given as a discrete number and unit of measurement
☐	☒	A statement on whether measurements were taken from distinct samples or whether the same sample was measured repeatedly
☐	☒	The statistical test(s) used AND whether they are one- or two-sided<br>*Only common tests should be described solely by name; describe more complex techniques in the Methods section.*
☒	☐	A description of all covariates tested
☒	☐	A description of any assumptions or corrections, such as tests of normality and adjustment for multiple comparisons
☐	☒	A full description of the statistical parameters including central tendency (e.g. means) or other basic estimates (e.g. regression coefficient) AND variation (e.g. standard deviation) or associated estimates of uncertainty (e.g. confidence intervals)
☐	☒	For null hypothesis testing, the test statistic (e.g. *F*, *t*, *r*) with confidence intervals, effect sizes, degrees of freedom and *P* value noted<br>*Give P values as exact values whenever suitable.*
☒	☐	For Bayesian analysis, information on the choice of priors and Markov chain Monte Carlo settings
☒	☐	For hierarchical and complex designs, identification of the appropriate level for tests and full reporting of outcomes
☒	☐	Estimates of effect sizes (e.g. Cohen's *d*, Pearson's *r*), indicating how they were calculated

Our web collection on statistics for biologists contains articles on many of the points above.

Software and code

Policy information about availability of computer code

Data collection	Images were acquired using STELLARIS 5 confocal microscope system (Leica), single-nucleus RNA-sequencing data were collected using NovaSeq 6000 sequencer, MERFISH data were collected using Vizgen Merscope instrument, Bulk RNA-sequencing data were collected using NextSeq 2000 P3.
Data analysis	snRNA-seq analysis: Cell Ranger (v6.0.0), R (v4.2.3), Seurat (5.1.0), Cellphone DB (v3.0), WGCNA (v1.7.3), scRFE (v1.5.6), enrichGO (v4.6.2)<br>MERFISH anlaysis: spyder (v3.9.14), numpy (v1.26.4), scanpy (v1.10.1), SpatialDE(v1.1.3), NaiveDE (v1.2.0), anndata (v0.10.7), pandas (v2.2.2);<br>Bulk RNA-seq analysis: STAR (2.7.10), DESeq2 (v1.38.3)<br>For imaging analysis, using Fiji (v2.0.0-rc-69/1,52p); for statistic analysis and plotting, using Prism 9; For astrocyte morphology analysis, using both Fiji (v2.0.0-rc-69/1,52p) and Imaris (v10.1). For more information see Methods.

For manuscripts utilizing custom algorithms or software that are central to the research but not yet described in published literature, software must be made available to editors and reviewers. We strongly encourage code deposition in a community repository (e.g. GitHub). See the Nature Portfolio guidelines for submitting code & software for further information.

Data

Policy information about availability of data

All manuscripts must include a data availability statement. This statement should provide the following information, where applicable:

- Accession codes, unique identifiers, or web links for publicly available datasets
- A description of any restrictions on data availability
- For clinical datasets or third party data, please ensure that the statement adheres to our policy

The datasets used in this research article can be downloaded from the Gene Expression Omnibus (GEO) accession number: snRNA-seq (GSE281738), MERFISH (GSE282127), Bulk RNA-Seq (GSE281480).
Publicly available data used in this study: GSE198027 (scRNA-seq of adult cortical, hippocampal and striatal astrocytes).

Research involving human participants, their data, or biological material

Policy information about studies with human participants or human data. See also policy information about sex, gender (identity/presentation), and sexual orientation and race, ethnicity and racism.

Reporting on sex and gender	No human research participants in the study.
Reporting on race, ethnicity, or other socially relevant groupings	No human research participants in the study.
Population characteristics	No human research participants in the study.
Recruitment	No human research participants in the study.
Ethics oversight	No human research participants in the study.

Note that full information on the approval of the study protocol must also be provided in the manuscript.

Field-specific reporting

Please select the one below that is the best fit for your research. If you are not sure, read the appropriate sections before making your selection.

☒ Life sciences ☐ Behavioural & social sciences ☐ Ecological, evolutionary & environmental sciences

For a reference copy of the document with all sections, see nature.com/documents/nr-reporting-summary-flat.pdf

Life sciences study design

All studies must disclose on these points even when the disclosure is negative.

Sample size	No statistical methods were used to pre-determine sample sizes but our sample sizes are similar to those reported in our previous publications (Xie, et al., 2022,Baizabal et al., 2018, Turrero-Garcia et al., 2021)
Data exclusions	All data was used for analysis unless apparent failures of the experiment.
Replication	The number of biological replicates used in snRNA-seq, MERFISH, bulk RNA-seq, immunostaining, in situ hybridization, HCR RNA-FISH and cell transplantation are shown in each figure legend and/of methods. For sequencing, we used all processed samples in the paper. The other experiments reported in this work were successfully repeated at a minimal of three times.
Randomization	Samples/organisms were allocated into experimental groups using a random allocation method. We used the same protocol for all samples to perform sequencing, immunostaining, in situ hybridization, HCR RNA-FISH, MERFISH, and bulk RNA-seq and cell transplantation.
Blinding	All samples were pooled to perform multiplexed snRNA-seq/MERFISH/bulk RNA-seq, so that different groups were processed in parallel and no human judgment was involved. For other experiments, sample collection was not fully performed blind to the experimental conditions, however, the data analyses were performed either using automated data analysis procedures without consideration of experimental groups or the experimenters were fully blind to the experimental conditions. And Data analysis was performed using the same parameters across groups.

Reporting for specific materials, systems and methods

We require information from authors about some types of materials, experimental systems and methods used in many studies. Here, indicate whether each material, system or method listed is relevant to your study. If you are not sure if a list item applies to your research, read the appropriate section before selecting a response.

April 2023

Materials & experimental systems

n/a	Involved in the study
☐	☒ Antibodies
☒	☐ Eukaryotic cell lines
☒	☐ Palaeontology and archaeology
☐	☒ Animals and other organisms
☒	☐ Clinical data
☒	☐ Dual use research of concern
☒	☐ Plants

Methods

n/a	Involved in the study
☒	☐ ChIP-seq
☒	☐ Flow cytometry
☒	☐ MRI-based neuroimaging

Antibodies

Antibodies used

Lhx2 (1:500-1000, rabbit, ABE1402, Millipore),
RFP (1:500, rabbit, 600-401-379, Rockland),
Sox9 (1:500, goat, AF3075, R&D),
Olig2 (1:2000, rabbit, AB9610, Millipore),
Zic (1:1000, rabbit, ref, gift from Segal lab, DFCI),
Nkx2.1 (1:500, clone-8G7G3/1, mouse, 53136, Santa Cruz),
GFP (1:500, Chicken, GFP-1020, Aves),
RFP (1:500, chicken, 600-901-379, Rockland),
PSD95 (1:500, rabbit, 51-6900, Thermo Fisher),
VGluT1 (1:500, guinea pig, AB5905, Millipore),
VGAT (1:500, clone-Gp117G4, guinea pig, 131308, Synaptic Systems),
Gephyrin (1:500, clone-RbmAb7a, rabbit, 147008, Synaptic Systems),
Calbindin (1:1000, rabbit, SKU CB38a, Swant),
Parvalbumin (1:500, clone-PARV-19, mouse, SAB4200545, Sigma),
Goat polyclonal anti-chicken Alexa 488, 546 (1:1000, A11039, A11040, Thermo Fisher),
Goat polyclonal anti-guinea pig Alexa 488 (1:1000, A11073, Thermo Fisher),
Goat polyclonal anti-mouse Alexa 488, 647 (1:1000, A11001, A21236, Thermo Fisher),
Goat polyclonal anti-rabbit Alexa 488, 647 (1:1000, A11034, A21245, Thermo Fisher),
Donkey polyclonal anti-goat Alexa 647 (1:1000, A21447, Thermo Fisher),
Donkey polyclonal anti-mouse Alexa 488 (1:1000, A21202, Thermo Fisher).

Validation

All other antibodies used in this study were obtained from commercial suppliers and were validated by the manufacturers for their application in immunohistochemistry. The validation is reported on their websites as follows:
Lhx2: https://www.sigmaaldrich.com/US/en/product/mm/abe1402
RFP-rabbit: https://www.rockland.com/categories/primary-antibodies/rfp-antibody-pre-adsorbed-600-401-379
Sox9: https://www.rndsystems.com/products/human-sox9-antibody_af3075
Olig2: https://www.sigmaaldrich.com/US/en/product/mm/ab9610
Nkx2.1: https://www.scbt.com/p/ttf-1-antibody-8g7g3-1
GFP: https://www.antibodiesinc.com/products/anti-green-fluorescent-protein-antibody-gfp
RFP-chicken: https://www.rockland.com/categories/primary-antibodies/rfp-antibody-600-901-379
PSD95: https://www.thermofisher.com/antibody/product/PSD-95-Antibody-Polyclonal/51-6900
VGLUT1:https://www.sigmaaldrich.com/US/en/product/mm/ab5905
VGAT:https://sysy.com/product/131308
Gephyrin: https://sysy.com/product/147008
Calbindin: https://webshop.swant.com/cb38a-calbindin.html
Parvalbumin: https://www.sigmaaldrich.com/US/en/product/sigma/sab4200545
Goat polyclonal anti-chicken Alexa 488: https://www.thermofisher.com/antibody/product/Goat-anti-Chicken-IgY-H-L-Secondary-Antibody-Polyclonal/A-11039
Goat polyclonal anti-chicken Alexa 546: https://www.thermofisher.com/antibody/product/Goat-anti-Chicken-IgY-H-L-Secondary-Antibody-Polyclonal/A-11040
Goat polyclonal anti-guinea pig Alexa 488: https://www.thermofisher.com/antibody/product/Goat-anti-Guinea-Pig-IgG-H-L-Highly-Cross-Adsorbed-Secondary-Antibody-Polyclonal/A-11073
Goat polyclonal anti-mouse Alexa 488: https://www.thermofisher.com/antibody/product/Goat-anti-Mouse-IgG-H-L-Cross-Adsorbed-Secondary-Antibody-Polyclonal/A-11001
Goat polyclonal anti-mouse Alexa 647:https://www.thermofisher.com/antibody/product/Goat-anti-Mouse-IgG-H-L-Highly-Cross-Adsorbed-Secondary-Antibody-Polyclonal/A-21236
Goat polyclonal anti-rabbit Alexa 488: https://www.thermofisher.com/antibody/product/Goat-anti-Rabbit-IgG-H-L-Highly-Cross-Adsorbed-Secondary-Antibody-Polyclonal/A-11034
Goat polyclonal anti-rabbit Alexa 647:https://www.thermofisher.com/antibody/product/Goat-anti-Rabbit-IgG-H-L-Highly-Cross-Adsorbed-Secondary-Antibody-Polyclonal/A-21245
Donkey polyclonal anti-goat Alexa 647: https://www.thermofisher.com/antibody/product/Donkey-anti-Goat-IgG-H-L-Cross-Adsorbed-Secondary-Antibody-Polyclonal/A-21447
Donkey polyclonal anti-mouse Alexa 488: https://www.thermofisher.com/antibody/product/Donkey-anti-Mouse-IgG-H-L-Highly-Cross-Adsorbed-Secondary-Antibody-Polyclonal/A-21202
In addtion, the Zic antibody is a Gift from Segal Lab, DFCI (Borghesani et al.,2002, DOI: 10.1242/dev.129.6.1435)

Animals and other research organisms

Policy information about studies involving animals; ARRIVE guidelines recommended for reporting animal research, and Sex and Gender in Research

Laboratory animals	Mouse lines: CD1 (strain code 022, Charles River Laboratories), C57BL/6J-Tg(Nkx2-1-cre)2Sand/J (Nkx2.1-Cre) (008661, The Jackson Laboratory), B6;129-Gt(ROSA)26Sortm5(CAG-Sun1/sfGFP)Nat/J (021039, The Jackson Laboratory), Zic4Cre (Kessaris lab, UCL [32]), B6.Cg-Gt(ROSA)26Sortm14(CAG-tdTomato)Hze/J (Ai14) (007914, The Jackson Laboratory), Tg(Slc1a3-cre/ERT)1Nat/J (Glast-CreERT2) (012586, The Jackson Laboratory), B6N.129-Ptch1tm1Hahn/J (Ptch1flox/flox) (012457, The Jackson Laboratory), Tg(Aldh1l1-EGFP,-DTA)D8Rth/J (026033, The Jackson Laboratory). the different lines at different ages were used based on different experiments. The detailed information is shown in figure legends and methods. Animals were housed in a room with the humidity between 30-70%; temperature 68-79 degrees F. and a 12 h light–dark cycle (19:00–07:00 schedule), ventilation maintained at 10-15 air changes per hour.
Wild animals	No wild animals were used in this study.
Reporting on sex	For snRNA-seq and MERFISH, sex was determined prior to use, but this was only applied to mice older than P7. Before P7, sex is difficult to distinguish in the first postnatal week, so these mice were labeled as sex-undetermined. For other experiments, results/data were not separated based on sex
Field-collected samples	No field-collected samples were used in this study
Ethics oversight	All animal procedures conducted in this study followed experimental protocols approved by the Institutional Animal Care and Use Committee of Harvard Medical School (HMS) (IS00000677-3)and the University of California, San Francisco (UCSF)(AN191974, AN205156-00B). Mouse housing and husbandry conditions were performed in accordance with the standards of the Center of Comparative Medicine at HMS and the Laboratory Animal Resource Center (LARC) at UCSF.

Note that full information on the approval of the study protocol must also be provided in the manuscript.

Plants

Seed stocks	*Report on the source of all seed stocks or other plant material used. If applicable, state the seed stock centre and catalogue number. If plant specimens were collected from the field, describe the collection location, date and sampling procedures.*
Novel plant genotypes	*Describe the methods by which all novel plant genotypes were produced. This includes those generated by transgenic approaches, gene editing, chemical/radiation-based mutagenesis and hybridization. For transgenic lines, describe the transformation method, the number of independent lines analyzed and the generation upon which experiments were performed. For gene-edited lines, describe the editor used, the endogenous sequence targeted for editing, the targeting guide RNA sequence (if applicable) and how the editor was applied.*
Authentication	*Describe any authentication procedures for each seed stock used or novel genotype generated. Describe any experiments used to assess the effect of a mutation and, where applicable, how potential secondary effects (e.g. second site T-DNA insertions, mosiacism, off-target gene editing) were examined.*
